# The complete 12 Mb genome and transcriptome of *Nonomuraea gerenzanensis* with new insights into its duplicated “magic” RNA polymerase

**DOI:** 10.1038/s41598-016-0025-0

**Published:** 2016-12-21

**Authors:** Valeria D’Argenio, Mauro Petrillo, Daniela Pasanisi, Caterina Pagliarulo, Roberta Colicchio, Adelfia Talà, Maria Stella de Biase, Mario Zanfardino, Emanuela Scolamiero, Chiara Pagliuca, Antonio Gaballo, Annunziata Gaetana Cicatiello, Piergiuseppe Cantiello, Irene Postiglione, Barbara Naso, Angelo Boccia, Miriana Durante, Luca Cozzuto, Paola Salvatore, Giovanni Paolella, Francesco Salvatore, Pietro Alifano

**Affiliations:** 1 CEINGE-Biotecnologie Avanzate, Naples, Italy; 2 Department of Molecular Medicine and Medical Biotechnology, Federico II University Medical School, Naples, Italy; 3 European Commission, Joint Research Centre (JRC), Ispra, Italy; 4 Department of Biological and Environmental Sciences and Technologies (DiSTeBA), University of Salento, Lecce, Italy; 5 Department of Biotechnology and Life Sciences, University of Insubria, Varese, Italy; 6 Department of Sciences and Technologies, University of Sannio, Benevento, Italy; 7 CNR NANOTEC – Institute of Nanotechnology, Center of Nanotechnology c/o Campus Ecotekne, Lecce, Italy; 8 CNR – Institute of Sciences of Food Production (ISPA), Operative Unit of Lecce, Lecce, Italy

## Abstract

In contrast to the widely accepted consensus of the existence of a single RNA polymerase in bacteria, several actinomycetes have been recently shown to possess two forms of RNA polymerases due the to co-existence of two *rpoB* paralogs in their genome. However, the biological significance of the *rpoB* duplication is obscure. In this study we have determined the genome sequence of the lipoglycopeptide antibiotic A40926 producer *Nonomuraea gerenzanensis* ATCC 39727, an actinomycete with a large genome and two *rpoB* genes, i.e. *rpoB(S)* (the wild-type gene) and *rpoB(R)* (the mutant-type gene). We next analyzed the transcriptional and metabolite profiles in the wild-type gene and in two derivative strains over-expressing either *rpoB(R)* or a mutated form of this gene to explore the physiological role and biotechnological potential of the “mutant-type” RNA polymerase. We show that *rpoB(R)* controls antibiotic production and a wide range of metabolic adaptive behaviors in response to environmental pH. This may give interesting perspectives also with regard to biotechnological applications.

## Introduction

Actinomycetes are ecologically important microorganisms that hold a prominent position as targets in screening programs due to their ability to produce a wide range of bioactive metabolites of industrial interest^1^. They are also unique amongst bacteria in their mycelial, sporulating life cycle, which involves complex regulation of gene expression in both space and time^1^. Actinomycetes are conventionally classified in two major groups: streptomycetes and “rare” actinomycetes. The latter term refers to strains whose isolation frequency is much lower than that of the streptomycete strains by conventional methods. Compared to the streptomycetes, rare actinomycetes show slower growth, more complex nutritional requirements, poorer sporulation and instability toward preservation.

The genus *Nonomuraea* is a rare actinomycete taxon with a long taxonomic history, while its generic description was recently emended^2^. The genus presently comprises more than 30 species that are widely distributed in soil, freshwater and marine environments with several strains recently isolated from acidic soils, rhizosphere, phyllosphere, coastal sediments and extreme or very changeable environments such as sand dunes and mangroves. Beside their ecological role, the genus *Nonomuraea* has a great potential for biotechnological applications. A broad range of potent bioactive compounds including antimicrobial, anticancer, and antipsychotic substances, and a broad spectrum of antibiotics and biocatalysts can be synthesized by the genus^2^. Notwithstanding these perspectives, genomic information about the genus *Nonomuraea* is, at present, still limited. Only a draft genome sequence of the myxochelin A producer *Nonomuraea* sp. TP-A0861 was published^3^, and the draft genomes of *Nonomuraea candida* NRRL B-24552 (JOAG00000000.1), *Nonomuraea coxensis* DSM 45129 (ARBV00000000.1), and *Nonomuraea kuesteri* NRRL B-24325 (JOAM00000000.1) were released to the public.

Here we present the complete genome sequence of *Nonomuraea gerenzanensis* ATCC 39727, an industrially important microorganism that was isolated from Indian soil^4^. This microorganism is the producer of the teicoplanin-like glycopeptide A40926 with anti*-Neisseria* activity^5^, which is the precursor of dalbavancin, a novel second-generation lipoglycopeptide antimicrobial with unique pharmacokinetics and excellent activity against resistant gram-positive pathogens, including methicillin-resistant *Staphylococcus aureus* ^6^. A peculiar feature of this microorganism is the presence of two types of RNA polymerases (RNAP). This is due to co-existence in its genome of two RNAP β chain-encoding genes: *rpoB(S)* (the wild-type *rpoB* gene) and *rpoB(R)* (a mutant-type *rpoB* gene)^7^. With respect to the *rpoB(S)* gene product, the product of *rpoB(R)* is characterized by six amino acid deletions in a hyper-variable region of the β lobe domain, and five amino acid substitutions located in the RNAP fork domain. This domain, which undergoes major conformational changes during the switch of RNAP from initiation to elongation mode^8^, is targeted by antibacterial compounds^9^. Two out of the five amino acid substitutions, i.e. a histidine-to-asparagine substitution (H526N in *E. coli* numbering) in the *rif* cluster I and a serine-to-tyrosine substitution (S531Y in *E. coli*) in the *rif* cluster II were associated to resistance to rifamycins and sorangicin, respectively^7^, and were also found in the corresponding regions of *rpoB2* from *Nocardia farcinica* IFM 10152, an actinomycete that shares with *N. gerenzanensis* ATCC 39727 the distinction of having two *rpoB* paralogs^10^.

The presence of both wild-type and mutant-type *rpoB* genes in the same genome may represent an elaborate strategy enabling certain actinomycetes to cohabit with microorganisms that produce antibiotics targeting the bacterial RNAP, minimizing, at the same time, the fitness cost often associated with antibiotic-resistance. However, the more intriguing possibility is that *rpoB* duplication may contribute to the developmental strategy of these bacteria. This hypothesis is supported by the evidence that *Nonomuraea terrinata* strains with duplicated *rpoB* genes exhibited *in vitro* much greater capability than single *rpoB* strains for growth, sporulation and antibiotic production under stressful conditions^11^. Moreover, *rpoB(R)* markedly activated antibiotic biosynthesis in the wild-type *Streptomyces lividans* strain 1326, and also in strain KO-421, a “relaxed” mutant unable to produce the regulatory nucleotide guanosine tetraphosphate (“magic spot”), and the RpoB(R)-specific histidine-to-asparagine substitution was essential for the activation of secondary metabolism by mimicking a “stringent” phenotype^11^. From a practical point of view, heterologous expression of *rpoB(R)* in desired actinomycetes can be exploited to boost production of secondary metabolites^12–14^. In this study with the aim to gain new insights about the physiological role of the “mutant-type” RNAP we have also determined the transcriptional profile in the wild type and in two derivative strains over-expressing either *rpoB(R)* or a retro-mutated (asparagine-back to-histidine codon) form of this gene.

## Results

### Genome anatomy of *N. gerenzanensis* ATCC 39727

**Figure 1:**
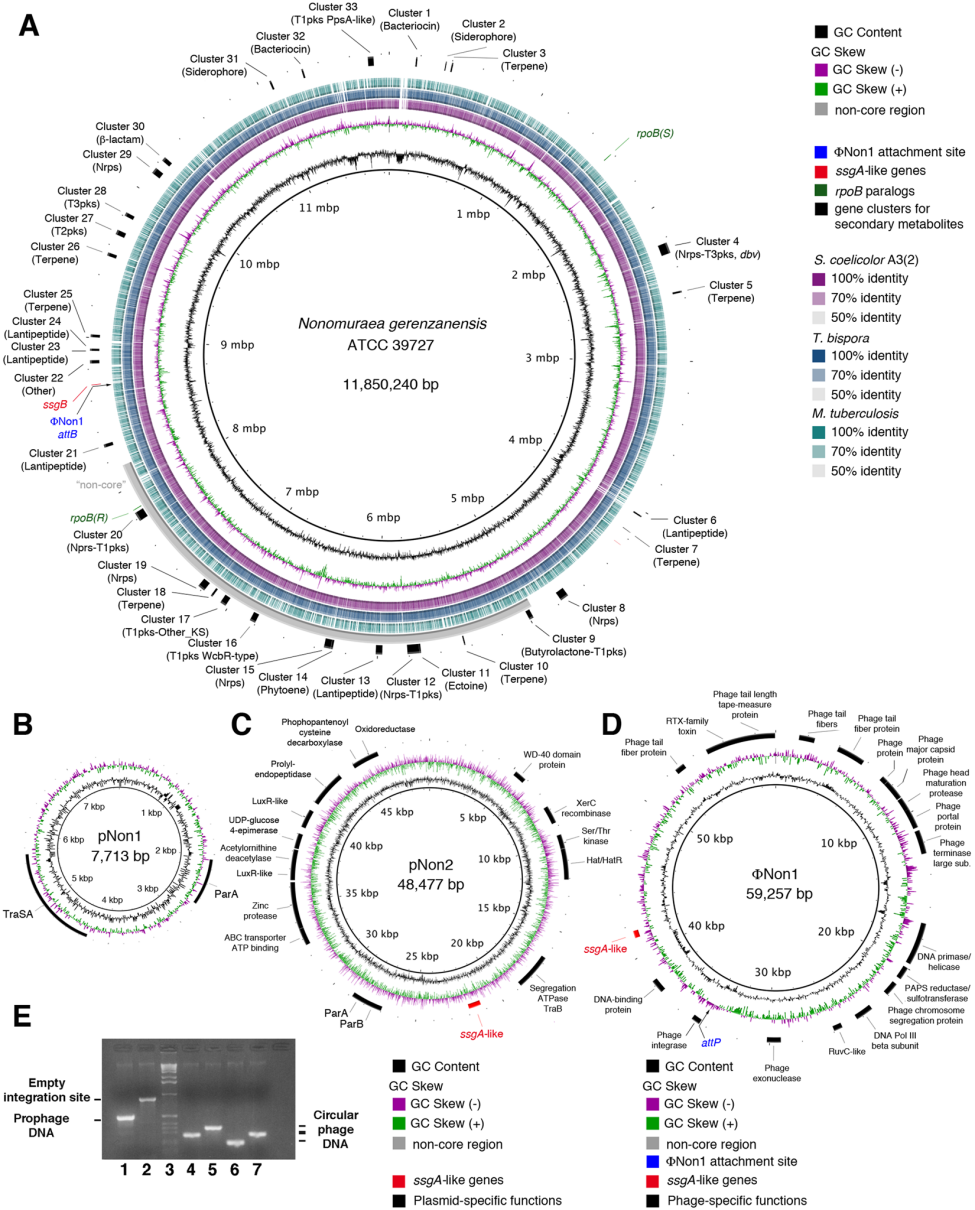
Schematic representation of the circular chromosome of *N. gerenzanensis*, and maps and features of the extra-chromosomal elements. (**A–D**) Representations of the chromosome (**A**), plasmids pNon1 (**B**) and pNon2 (**C**), and the plasmid-phage element ΦNon1 (**D**) were obtained by the Blast Ring Image Generator (BRING). In panel *A* the *N. gerenzanensis* ATCC 39727 chromosome sequence was aligned with those of reference actinobacteria *S. coelicolor* A3(2), *Mycobacterium tuberculosis* H37Rv and *T. bispora* DSM 43833 as shown. In addition to GC content and GC skew, location of the rRNA operons, the gene clusters coding for secondary metabolites, the two *rpoB(S)* and *rpoB(R)* paralogs, *ssgB* (*ssgA*-like) gene, and the ΦNon1 attachment site are shown in panel *A*. The grey arc marks position of the “non-core” region. In panels B–D location of several phage/plasmid elements is shown including the *ssgA*-like genes in pNon2 and ΦNon1 (**C,D**). (**E**) Chromosomally integrated prophage ΦNon1 DNA (lane 1), empty chromosome *attB* integration site (lane 2) and circular ΦNon1 DNA (lanes 4–7) detected by PCR with specific primers.

Ten GS20 runs, 1 GS FLX and 1 GS FLX Titanium run were totally performed, as described under online methods, to ensure high sequencing coverage depth on the entire *N. gerenzanensis* ATCC 39727 genome. Two paired-end Titanium libraries (3 and 8 kb, respectively) were also sequenced to obtain oriented genomic contigs and support genome assembly. Finally, we used a PCR-based strategy to fill-in gaps between contigs^15, 16^. The final genome resulted of about 12 Mb in size organized in one main chromosome and three extra-chromosomal elements (Fig. 1 and Table S1A). The genome sequence revealed considerable coding potential with 11,057 CDSs, and a large percentage of genome (13.5%) devoted to regulation, particularly transcription regulation (Table S1B). SEED-Viewer analysis with the sequences available at the RAST server suggested that the closest neighbors of *N. gerenzanensis* ATCC 39727 were *Thermobispora bispora* DSM 43833 and *Streptosporangium roseum* DSM 43021. Analysis of the relative ortholog positions in the chromosome sequence of *N. gerenzanensis* and in the assembled chromosome sequences of *T. bispora* and *S. roseum* (Figure S1) revealed extensive synteny on a genome-wide scale except for a 3.2 Mb region (indicated as “non-core” in Fig. 1A) that was enriched in gene clusters coding for secondary metabolites and their predicted precursors, together with a large number of genes involved in microbial adaptation under stressful conditions (Table S1B). Some of these “contingency” genes codes for anaerobic respiratory reductases, high-affinity transport systems, or proteins involved in cellulose, aromatic compound and alkane sulfonate metabolism, antibiotic and heavy metal resistance, and pH homeostasis (Na^+^/H^+^ antiporters, arginine deiminase pathway).

The extra-chromosomal elements consisted of a small plasmid, pNon1 (7,713 bp, 66.0% GC, copy number 5) (Fig. 1B), a large plasmid, pNon2 (48,477 bp, 69.8% GC, copy number 4) (Fig. 1C), and a large self-replicating element, ΦNon1 (59,257 bp, 70.0% GC, copy number 17) (Fig. 1D), harboring a chimeric prophage combining structural genes from distinct phage families. ΦNon1 is reminiscent of pZL12/ΦZL12, a *Streptomyces* spp. plasmid-phage element^17^. ΦNon1 was detected by PCR and DNA sequencing as either circular phage DNA or prophage DNA (Fig. 1E), integrated, at high frequency, into the host chromosome at a specific 50 bp-long *attB* site (Fig. 1A,D) overlapping the 3′-end of a tRNA^Val (CAC)^ gene. Phage integration, which does not disrupt the integrity of the tRNA gene, occurs in close proximity to an *ssgA*-family morphogene (*ssgB*). SsgA-like proteins are a family of homologous cell division-related proteins that occur in morphologically complex actinomycetes^18^. Notably, two additional *ssgA*-family morphogenes were mapped, the first one in ΦNon1 (Fig. 1D) and the other one in pNon2 DNA (Fig. 1C) suggesting an involvement of the extra-chromosomal elements in morphological differentiation.

Paralogous gene expansion is a notable trait of *N. gerenzanensis* (Table S1C). In addition to *rpoB* ^7^, a plethora of genes involved in basic cell processes such as chromosome replication, DNA repair, cell division, translation, transcription, central intermediary and energy metabolism (respiratory chain, ATP synthase) were duplicated or further expanded. As genome expansion represents an increased cost for most organisms, a crucial question to ask is what is the evolutionary driving force behind retention of duplicated genes. The genome features of *N. gerenzanensis* make it a suitable model organism to investigate on this subject.

### Secondary metabolism: the hidden chemical treasure

**Figure 2:**
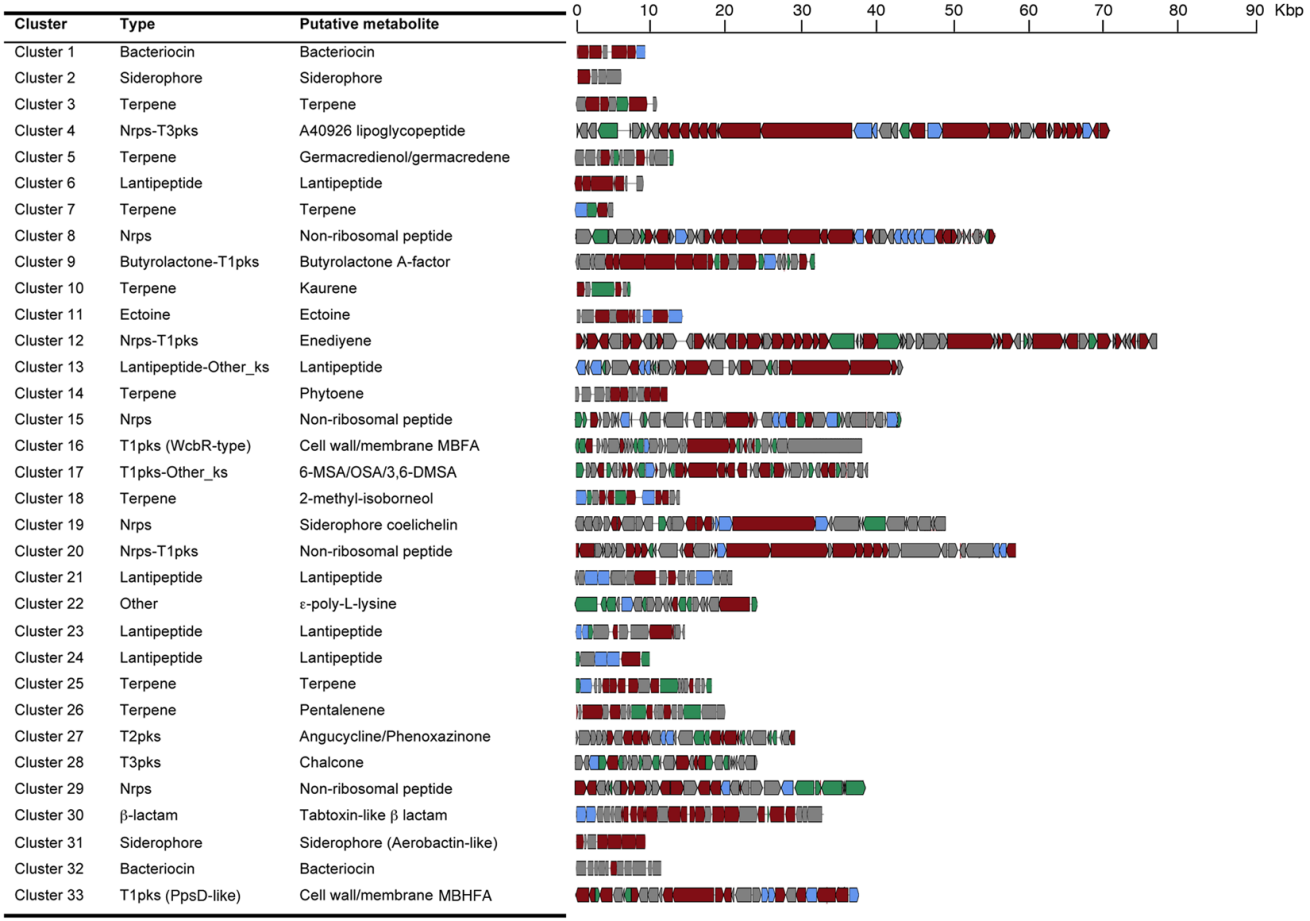
Overview of the gene clusters coding for secondary metabolites as inferred from analysis of the*N. gerenzanensis* genome by the antiSMASH platform. Abbreviations: t1pks, type I polyketide synthase; t2pks, type II polyketide synthase, t3pks, type 3 polyketide synthase; nrps, non-ribosomal peptide synthase; hglks, heterocyst-type glycolipid ketosynthase; pls, ε-poly-L-lysine synthase; 6-MSA, 6-methyl salicylic; 3,6-DMSA 3,6-dimethyl salicylic; OSA, orsellinic acid; MBFA methyl-branched fatty acid; MBHFA, methyl-branched-hydroxylated fatty acid; PUFA, polyunsaturated fatty acid.

*N. gerenzanensis* ATCC 39727 is known for its ability to produce the lipoglycopeptide antibiotic A40926 whose gene cluster (*dbv*) (cluster 4 in Fig. 2 and Table S1D) was characterized more than 10 years ago^19^. Genome mining with antiSMASH indicated the presence of further 32 clusters governing the synthesis of polyketides (Type 1, 2 and 3 Pks clusters; abbreviated T1pks, T2pks, T3pks), non-ribosomally synthesized peptides (Nrps clusters), lantipeptides and terpenes (Fig. 2 and Table S1D). As well as in other actinomycetes, the distribution of these clusters is not uniform around the chromosome: many of them (clusters 9 to 20) are located in the “non-core” region (Fig. 1A).

Computational methods allowed us to predict their putative metabolic products. Cluster 12 (Nrps-T1pks) would encode an enediyne (Fig. 2 and Figure S2). These compounds including maduropeptin, neocarzinostatin and C-1027 are considered nowadays very promising molecules in anticancer therapy^20, 21^. Enediyne compounds share some structural and functional similarities. One fragment of a structure is responsible for the recognition and transport, another part acts as molecular trigger while the third, reactive enediyne unit, undergoes Bergman cycloaromatization and causes DNA breakage^22^.

Cluster 27 (T2pks) would encode an unknown molecule with an angucycline core (Fig. 2 and Figure S3). These compounds show a multitude of valuable biological activities such as anticancer, antibacterial, antifungal and antiviral activity^23^. The angucycline polyketide backbone is generated by T2pks through condensation of an acetyl starter unit and multiple malonyl-CoA extender units. The backbone is then regiospecifically folded and cyclised by aromatase and cyclase enzymes, and finally modified by tailoring reactions to yield the final molecule. The presence in this cluster of two phenoxazinone synthases (PHS)-encoding genes suggests that these enzymes may be involved in decoration of the angucycline core. PHSs are multi-copper oxidases that participate in spore pigmentation and antibiotic biosynthesis^24^.

Of the remaining Pks gene clusters (Fig. 2), cluster 16 codes for a WcbR-type T1pks that appears to govern the biosynthesis of a methyl-branched fatty acid that in other bacteria seems to be involved in plant- and sponge-microbe interactions^25, 26^. Cluster 17 would encode an iterative T1pks apparently involved in 6-methyl salicylic/3,6-dimethyl salicylic/orsellinic acid biosynthesis, while cluster 33 coding for a PpsD-like T1pks (related to phtiocerol dimycoceroserate in *Mycobacterium* spp.) might be involved in biosynthesis of methyl-branched-hydroxylated fatty acid. Cluster 8 contains the entire set of genes coding for γ-butyrolactone (quorum sensor) biosynthetic enzymes.

Among the Nrps gene clusters (Fig. 2), cluster 20 is remarkably similar to hybrid Nrps-T1pks from *Serratia* spp. In silico analysis predicted that valine, arginine, malonate, cysteine and aspartic acid may be sequentially incorporated to form an unknown metabolite, which can be then modified by cluster-encoded dioxygenase and cysteine desulfurase. The presence in this cluster of both SyrP-like (iron-containing transcriptional regulator) and PhcC-like (thioesterase involved in pyochelin biosynthetis) -encoding genes suggests that it may govern the synthesis of a siderophore. Cluster 19 exhibits considerable homology to the Pks cluster devoted to the biosynthesis of coelichelin, the tripeptide siderophore of *S. coelicolor* A3(2). Cluster 22 codes for ε-poly-L-lysine synthase. ε-poly-L-lysine is produced by several actinomycetes when fermentation broth becomes acidic during the stationary growth phase^27^. Interestingly, cluster 30 is highly homologous to the gene cluster that controls the biosynthesis of tabtoxin β-lactam in phytopathogenic *Pseudomonas syringae* (Fig. 2 and Figure S4)^28^. Tabtoxin irreversibly inhibits the enzyme glutamine synthetase causing cells to become intoxicated by high levels of their own unprocessed ammonia causing chlorosis in plants^29^.

Among the terpene synthase gene clusters (Fig. 2), cluster 18 exhibits the typical structure of clusters involved in the biosynthesis of the odorous compound 2-methyl-isoborneol. Cluster 5 contains a CDS displaying highest homology with the first sesquiterpene domain of germacredienol/germacredene D synthase, while cluster 26 includes genes coding for a pentalenene synthases. Clusters 10 and 14 contain genes related to those coding for kaurene synthase and phytoene synthase, respectively (Fig. 2). Five lantipeptide gene clusters were also identified. For three of them putative lantipeptide structures were predicted (Figures S5–7).

### The transcriptome: rpoB duplication and the global metabolic switch

**Figure 3:**
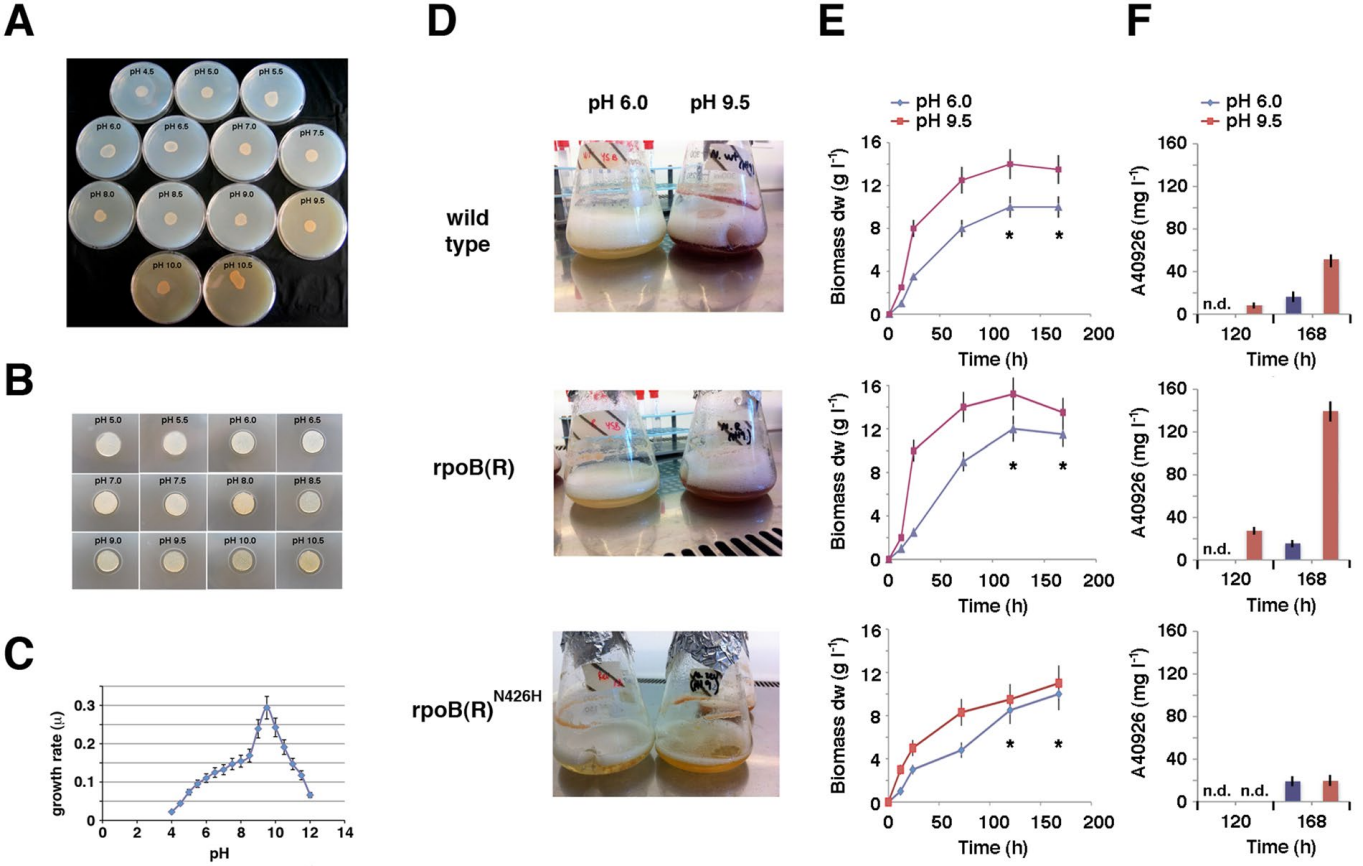
Growth, phenotype and antibiotic production at different pH. (**A**,**B**) Pictures showing growth (**A**) antibiotic production (**B**) of *N. gerenzanensis* grown on YS agar at different initial pH values. Antibiotic production was evaluated by microbiological assay using *S. aureus* as a tester microorganism. (**C**) Specific growth rates (μ = number of replication per h) during the logarithmic phase of *N. gerenzanensis* growing in YS broth at different pH values. Data are shown as mean ± standard deviation from at least three independent experiments. (**D**–**F**) Red pigment and A40926 antibiotic production in response to pH by *N. gerenzanensis* and derivative rpoB(R) and rpoB(R)^N462H^ strains. In panel **D** flasks containing *N. gerenzanensis* and derivative strains after growth in liquid YS broth at pH 6.0 and 9.5 for 168 h are shown. Biomass and A40926 production by *N. gerenzanensis* and derivative strains growing in YS broth at pH 6.0 and 9.5 are shown, respectively, in panels *E* and *F*. A40926 production was evaluated by HPLC. Data are shown as mean ± standard deviation from at least three independent experiments.

A distinctive feature of *N. gerenzanensis* ATCC 39727 is the presence of duplicated polymorphic *rpoB* paralogs^7^. The ability of *N. gerenzanensis* to grow within a wide range of pH values from about 4.0 to 12.0, and hyper-produce antibiotic at high pH values (Fig. 3A–C) prompted us to investigate the existence of a possible interplay between environmental pH, *rpoB(R)* expression and antibiotic production. We focused on pH because it exerts profound effects on bacterial physiology, metabolism and gene expression^30^, and it is one of the most important environmental factors affecting antibiotic production by actinomycetes^31–36^. Moreover, pH shifts have been shown to induce the stringent response in several bacteria^37, 38^.

As attempts to obtain either *rpoB(R)* knockout mutants or gene replacement mutants, i.e., *rpoB(S)* or *rpoB(R)* by *rpoB(R)* or *rpoB(S*), were not successful, an approach based on introduction of additional wild type or mutated *rpoB(R)* copies was pursued. Two derivative strains were created. The first derivative strain, *N. gerenzanensis* • pTYM-rpoB(R), indicated hereinafter as rpoB(R) strain -, harbors additional copies of *rpoB(R)*. It was obtained by using a gene transfer system based on intergeneric conjugation from *Escherichia coli* and integration via ΦC31 recombinase (Figure S8). In the second strain, *N. gerenzanensis* • pTYM-rpoB(R)^N426H^ - indicated hereinafter as rpoB(R)^N426H^ strain -, the asparagine codon of the additional *rpoB(R)* copy was mutated back to the histidine codon that is present in *rpoB(S)* and, normally, in bacterial wild-type *rpoB* genes. This back mutation abrogated rifampicin-resistance and activation of antibiotic biosynthesis by *rpoB(R)* in *S. lividans* ^11^ *.* In the rpoB(R)^N426H^ strain the mutated *rpoB(R)* ^N426H^ allele was expected to compete with the endogenous *rpoB(R)* allele.

Figure 3D–F and Figure S9 show that wild type and rpoB(R) strains exhibit similar phenotypes, with both pigmentation and A40926 antibiotic production being greatly stimulated at higher pH values (pH 9.5 vs. pH 6.0) in yeast starch (YS) medium, although higher antibiotic titers were reached in rpoB(R) than in wild type strain. In contrast, both pigmentation and A40926 antibiotic production were markedly reduced in rpoB(R)^N426H^ strain at alkaline pH. These results confirmed the involvement of *rpoB(R)* in the control of A40926 antibiotic production^7^, and the importance of the asparagine codon^11^, suggesting a possible link between *rpoB(R)* expression, biochemical and morphological differentiation and environmental pH.

**Figure 4:**
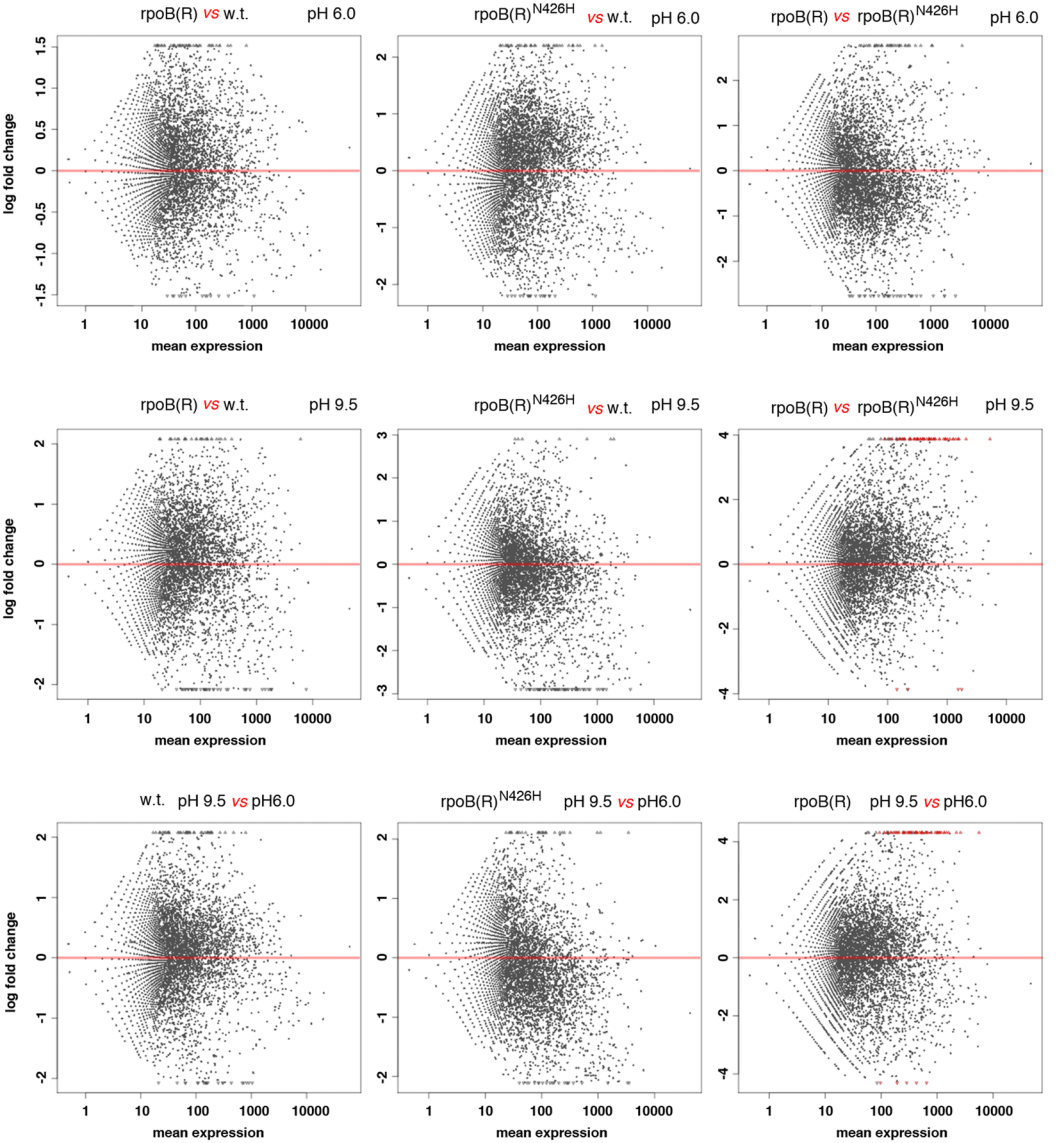
Differential expression analysis. MA-plots of normalized mean expression versus log_2_ fold change for the indicated sample pairs as inferred from RNA-Seq data. y-axis: log2 fold changes. x-axis: mean of normalized counts.

This cue was explored by comparative transcriptomic analysis. The Illumina RNA-Seq technology was used to examine the expression profiles of the wild type *N. gerenzanensis* and the derivative rpoB(R) and rpoB(R)^N426H^ strains during the late exponential growth (120 h) in slightly acidic (pH 6.0) or alkaline (pH 9.5) YS medium. In Fig. 4, RNA-Seq raw data (Table S2) are reported as scatter plots of log_2_ of fold change [y-axis] versus mean of normalized counts [x-axis] (MA-plots by *DESeq2* package)^39^. Gene-set enrichment analysis (GSEA) was performed to search for differences in the expression of groups of functionally related genes. To this aim, the GSEA tool was used to test for differential expression of 59 gene-sets, containing genes involved in central, intermediary and secondary metabolism (Table S3 and Table S4). Gene sets C_1–33_ correspond to the secondary metabolite clusters reported in Fig. 2; gene-sets D_1–23_ are groups of manually defined dispersed genes and include 22 groups of genes involved in central and intermediary metabolism, 3 groups containing genes located on the extra-chromosomal elements and 1 group of tRNA genes.

**Figure 5:**
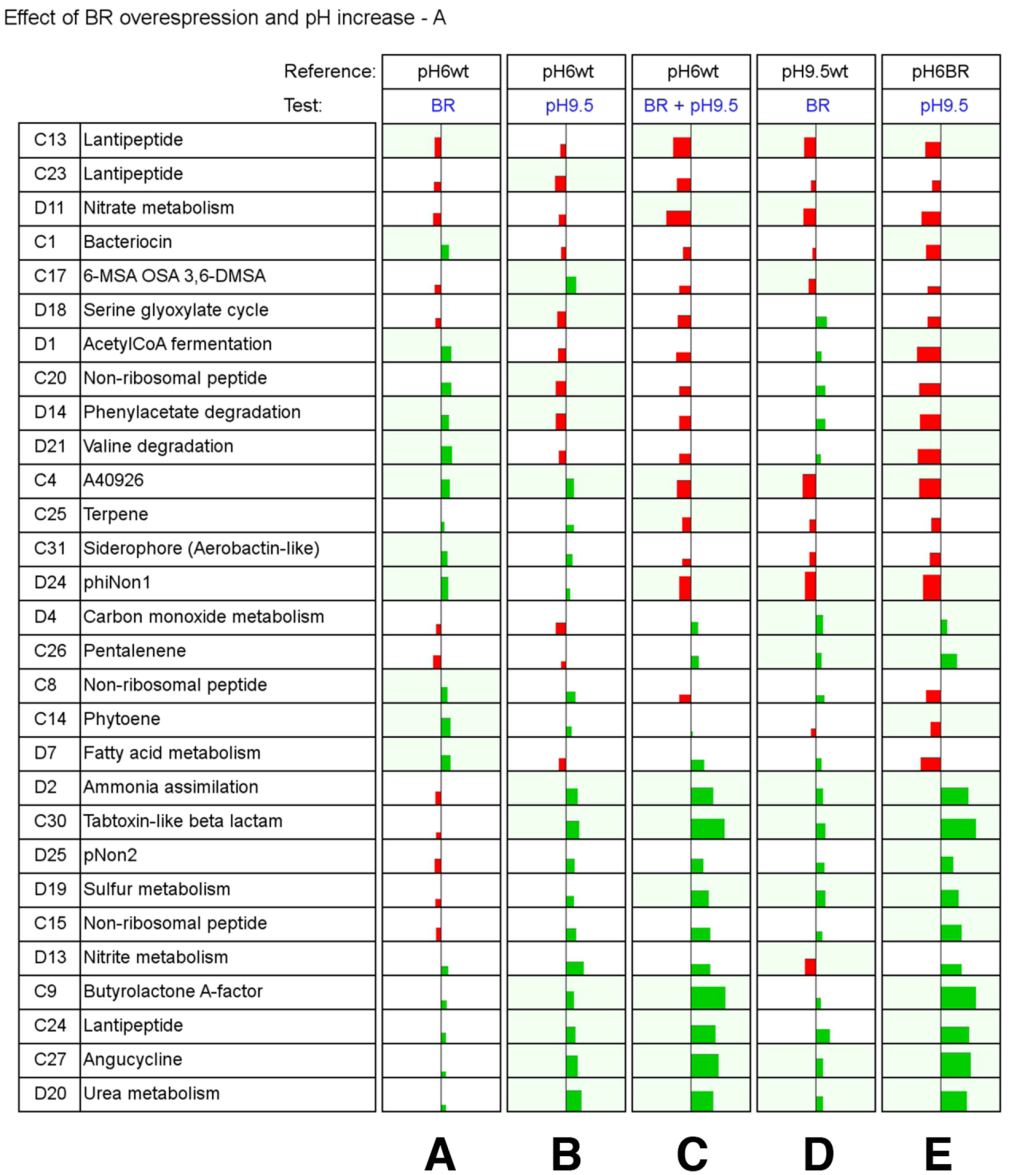
Overview of GSEA results. Effects of *rpoB(R)* over-expression and pH increase are reported by contrasting the rpoB(R) strain to wild type strain data. Gene-sets with Normalized Enrichment Score (NES) >1.70 and False Discovery Rate (FDR) <0.1 in at least one of the contrasts are reported. Green and red colors indicate, respectively, up-regulation and down-regulation in test strain vs. reference strain. If a set passed these thresholds in a contrast, the background of the cell is colored in pale green. For each set in each contrast, the width of the rectangle represents the mean log_2_FC of the leading edge subset, while the height represents the NES. Gene-sets are labeled with an ID indicating whether they consist in clustered (ID number preceded by the letter C) or dispersed (ID number preceded by the letter D) genes. Abbreviations: wt, wild type strain; BR, rpoB(R) strain.

Figure 5 reports GSEA the results obtained using a visualization method that allows to look at multiple sample comparisons in parallel. In Fig. 5 only gene-sets with a Normalized Enrichment Score (NES) >1.70 and a False Discovery Rate (FDR) <0.1 in at least one contrast are showed; Figures S10, 12, and 14 -contain gene-sets not passing the thresholds in any of the contrasts. This kind of visualization highlights parallel expression patterns and was used to reveal changes related to pH shift and addition of an exogenous copy of wild type *rpoB(R)* or a mutated *rpoB(R)* ^*N426H*^ gene. Several gene-sets appear to be synergistically up-regulated by alkaline pH and exogenous *rpoB(R)* addition (Fig. 5), including secondary metabolite gene clusters 27 (Angucycline/Phenoxazinone), 9 (Butyrolactone A-factor), 15 (Non-ribosomal peptide), 30 (Tabtoxin-like β-lactam), 24 (Lantipeptide morphogen), pNon2 genes, and gene-sets “ammonia assimilation”, “urea metabolism” and “sulfur metabolism”. Switching from pH 6 to 9.5 and introduction of a second copy of the *rpoB(R)* gene produce an interesting synergic effect, as the strong up-regulation observed in presence of both (Fig. 5C) is not observed for any of the two taken separately (Fig. 5A,B). This effect depends on an increased pH sensitivity of the rpoB(R) strain (Fig. 5E), which is specifically related to the H426N change, given that in its absence (rpoB(R)^N426H^ strain) it is completely lost (Figure S11C).

Another change of expression pattern was observed for a number of gene-sets down-regulated in response to alkaline pH and exogenous *rpoB(R)* or *rpoB(R)* ^*N426H*^ addition (Fig. 5 and Figure S11). These include gene clusters 13 and 23 (Lantipeptides), 1 (Bacteriocin), 17 (6-MSA/OSA/3,6-DMSA), 20 (Nonribosomal peptide), ΦNon1 genes, and gene-sets “nitrate metabolism”, “serine glyoxylate cicle”, “phenylacetate degradation”, “butanoate metabolism” and “valine degradation”. Interestingly, pathways “phenylacetate degradation” and “homogentisate pathway” exhibit the same trend, though below the threshold. These pathways are involved in degradation of aromatic amino acids that are used as building blocks in A40926 biosynthesis^19^. Down-regulation of these gene-sets in response to alkaline pH was thus not dependent on the specific H426N substitution in *rpoB(R).* Some of these gene-sets were up-regulated by exogenous *rpoB(R)* or *rpoB(R)* ^*N426H*^ addition at pH 6.0; others exhibited the opposite behavior.

**Figure 6:**
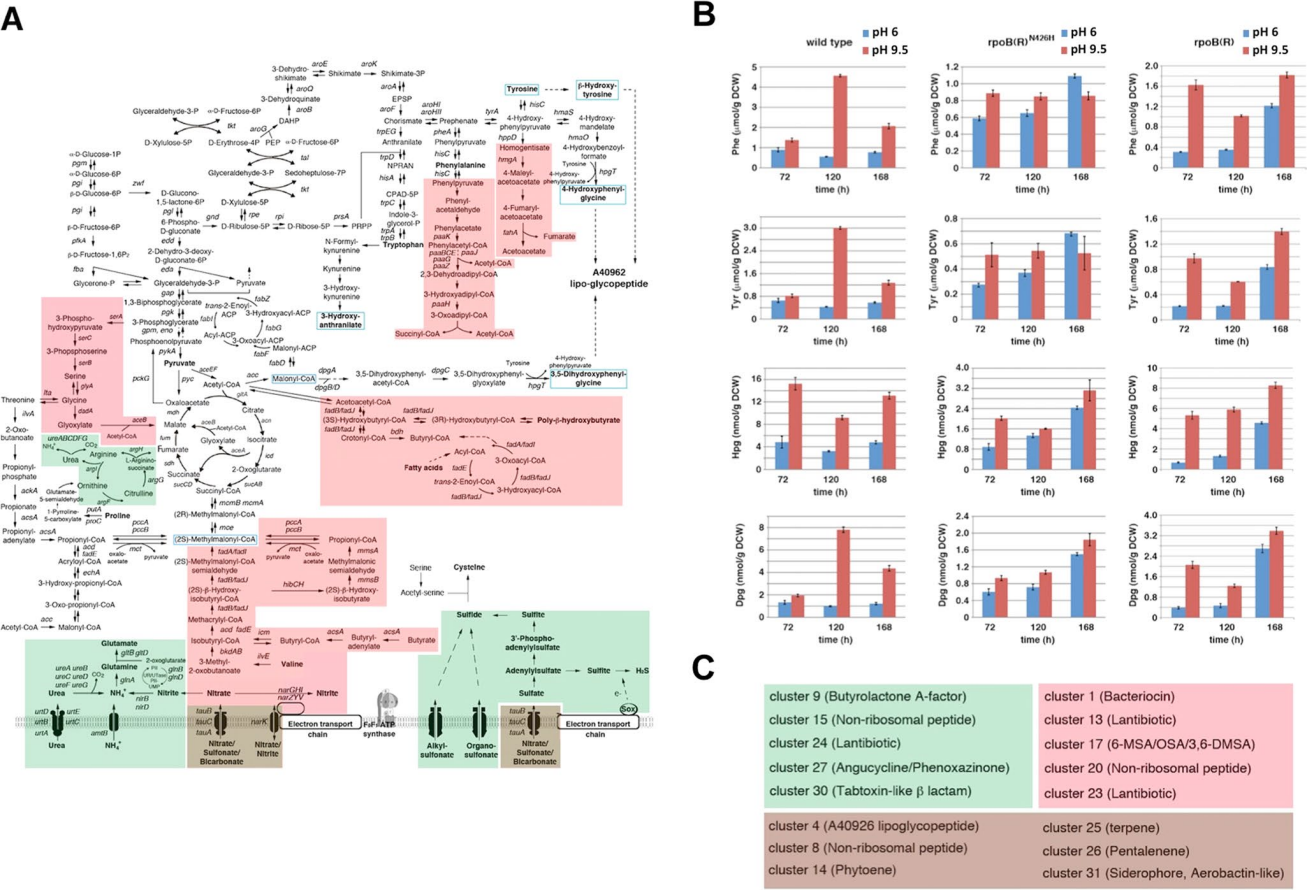
Metabolic pathways, gene clusters for secondary metabolites, and quantitative determination of intracellular levels of A40926 lipoglycopeptide biosynthetic precursors. (**A**) Metabolic pathways affected by environmental pH and *rpoB(R)* based on GSEA data. Green shading marks metabolic pathways whose gene-sets are synergistically up-regulated by alkaline pH and exogenous *rpoB(R)* addition. Red shading marks metabolic pathways whose gene-sets exhibit a trend of down-regulation in response to alkaline pH and exogenous *rpoB(R)* or *rpoB(R)* ^*N426H*^ addition. Brown shading identifies metabolic pathways whose gene-sets exhibit a more complex pattern depending on the different behavior of paralogs. (**B**) Quantitative assessment of intracellular levels of phenylalanine (Phe), tyrosine (Tyr), 3,5-dihydroxyphenylglycine (Dpg) and 4-hydroxyphenylglycine (Hpg) in wild type and derivative strains at different time points. (**C**) Gene clusters for secondary metabolites affected by environmental pH and *rpoB(R)* based on GSEA data. Green and red shadings are as in A. Brown shading identifies gene clusters with a more complex expression pattern.

More complex expression patterns were associated with gene clusters 4 (A40926 lipoglycopeptide), 25 (Terpene), 31 (Siderophore, Aerobactin-like), 26 (Pentalenene), 8 (Non-ribosomal peptide) and 14 (Phytoene), and gene sets “carbon monoxide metabolism” and “fatty acid metabolism”. In particular, the expression pattern of cluster 4 was not consistent with phenotypic data (Fig. 3). Indeed, the cluster was moderately up-regulated in the wild type but significantly down-regulated in the rpoB(R) strain in response to alkaline pH, whereas A40926 production was stimulated in both strains under these conditions. Compared to the wild type strain, this cluster was down-regulated at pH 9.5 and up-regulated at pH 6 in both the rpoB(R) and rpoB(R)^N426H^ strains (Fig. 5 and Figure S11). These data indicated that the effects of pH and exogenous *rpoB(R)* or *rpoB(R)* ^*N426H*^ gene copies on A40926 lipoglycopeptide production were not clearly associated with modulation of cluster 4 mRNA levels. Based on the expression patterns of the gene-sets “phenylacetate degradation” and “homogentisate pathway”, we hypothesized that A40926 production levels could be more closely associated with the levels of its biosynthetic precursors. This hypothesis was proven to be correct by quantitative assessment of intracellular levels of phenylalanine, tyrosine, 3,5-dihydroxyphenylglycine and 4-hydroxyphenylglycine in wild type and derivative strains at different time points (Fig. 6A,B). HPLC analysis demonstrated that the intracellular concentrations of these compounds in both wild type and rpoB(R) strains were significantly higher at pH 9.5 than at pH 6. In contrast, in the rpoB(R)^N426H^ strain, the levels of these molecules were not affected by environmental pH, and, at pH 9.5, were significantly lower than those of the other two strains.

## Discussion

In addition to identifying the metabolic potential of a valuable microorganism, this study is one of the first to explore the physiological and genetic responses of an actinomycete to environmental pH. In fact, despite the importance of the pH of the culture medium in industrial antibiotic fermentation^31, 32, 34, 35, 40^, in most reports, cell growth and antibiotic production by actinomycetes have been studied under near-neutral pH conditions. We provide evidence for a master regulatory function of the *rpoB* duplication (Fig. 6A,C). *rpoB(R)* plays a key role in the developmental strategy of *N. gerenzanensis* in harsh environments, in particular, where the bacteria have to procure sulfur and nitrogen for metabolism and macromolecular synthesis. In alkaline medium, *rpoB(R)* stimulates the expression of sulfur and nitrogen uptake- and metabolism-related genes as well as the production of a number of secondary metabolites. In contrast, it exerts an inhibitory effect on expression of a number of genes involved in central and intermediary carbon metabolism leading to increased availability of precursors for A40926 antibiotic biosynthesis.

The chemical-physical basis for the interplay between environmental pH and nitrogen regulon could probably be sought in the bioavailability of ammonium, which is a primary nitrogen source for most of soil bacteria and fungi. In soil ammonium forms a dynamic chemical equilibrium with ammonia. This equilibrium is very dependent on pH, and shifts to ammonia when the pH is high (9.0 and higher) causing ammonia volatilization and a net loss of nitrogen from soil. Indeed, in *N. gerenzanensis* high pH stimulates the expression of the ammonium transporter *amtB* gene along with other members of the nitrogen regulon. Up-regulation of cluster 15 may be part of this strategy because tabtoxin-like β-lactams inhibit the glutamine synthetase of organisms competing for ammonium. The strict interplay between nitrogen and secondary metabolism is in agreement with previous data^7, 41, 42^.

Up-regulation of the sulfur uptake- and metabolism-related gene-set (the so-called *cys* regulon) in an alkaline environment is instead consistent with previous results obtained in *Shewanella oneidensis* ^43^, and may be due to inhibition of sulfate uptake at alkaline pH. In most known bacteria, synthesis of biologically important sulfur containing molecules such as cysteine, methionine, *S*-adenosylmethionine (SAM) and antioxidant glutathione, mostly depends on sulfate transport into the cell and reductive sulfate assimilation. Although sulfate can gain entrance into bacterial cell by different types of transport systems^44^, in many environments with relatively low sulfate concentrations, sulfate uptake relies specifically on the high specificity transporter CysZ, and is driven by the proton gradient^45^. Indeed, in both plant and bacteria, sulfate uptake is pH dependent, can be abolished in the presence of either proton-translocating F_1_F_0_-ATPase inhibitors^46^ or proton ionophores^47^, and is inhibited at alkaline pH^45^. In fact, in alkaline environments (*N. gerenzanensis* is able to grow in an extremely wide pH range from 4 up to 12, Fig. 3C) the protonmotive force is lowered by the need to maintain a cytoplasmic pH well below the pH outside. It is reasonable that in these environments the reduction in sulfate uptake may stimulate the *cys* regulon that includes genes for uptake and utilization of alternative sulfur sources (Fig. 6A).

It is noteworthy that, in spite of the wide effects of *rpoB(R)* on overall gene expression in alkaline media, *rpoB(R)* mRNA levels did not change significantly in response to environmental pH. Of course, this finding does not exclude either the possibility of complex spatiotemporal regulation of this gene, or the existence of regulatory mechanisms operating at the level of RNA processing and/or translation in response to pH. Another intriguing issue concerns the mechanisms by which the “mutant-type” RNAP exerts its global effects on gene expression. The results in this study confirm the central role of the H526N substitution. Equivalent substitutions have been found in other actinomycetes that share with *N. gerenzanensis* the distinction of having two *rpoB* paralogs^10, 11^. Previous results indicate that mutant RNAPs with the same or similar substitutions behave as “stringent” RNA polymerases mimicking the effects of alarmone ppGpp and altering the transcription dynamics^11, 48, 49^. If we consider the well-known strict interplay between the stringent response and nitrogen metabolism^50–52^, the effects of *rpoB(R)* on the expression of nitrogen regulon seem to be consistent with this hypothesis, although this aspect clearly deserves further investigation.

In conclusion, after having analyzed and fully described one of the largest bacterial genomes and transcriptome sequences (~12 Mbase), we show that *rpoB* duplication oversees a global metabolic switch demonstrating that co-existence of “wild-type” and “mutant-type” RNAPs in several actinomycetes is a successful adaptative strategy to survive and thrive in harsh and rapidly evolving environments. This provides theoretical and methodological bases to boost production of desired compounds or uncover new metabolic capabilities by RNAP genetic engineering^12–14^, thus giving new perspectives for biotechnologically useful productive applications. Indeed, RNAP genetic engineering is proving to be a powerful technology for strain improvement and drug discovery through the activation of silent biosynthetic gene clusters^12–14, 53, 54^, and combination of this technology with pH management can further expand its potential in bacteria that have a hidden chemical treasure of compounds whose production is differentially affected by environmental pH.

## Methods

### Bacterial strains, growth conditions and metabolite assays

*Nonomuraea gerenzanensis* (emended classification of *Nonomuraea* sp. ATCC 39727) was obtained from the American Type Culture Collection (ATCC). The strain was originally classified as a member of the genus *Actinomadura* ^5^. Following a reclassification of the genera *Actinomadura* and *Microtetraspora* and a further reanalysis of the latter genus^55^, the A40926 producer was classified as a *Nonomuraea* species^56^, and recently as type strain of the novel species *N. gerenzanensis* ^4^. Compositions of the media used in this study (seed medium [SM], yeast starch [YS], and oat meal yeast [OMY]) were previously described^7^. The YS medium was used as basal medium to analyze the effects of pH (which was adjusted to the desired values using HCl or NaOH).

In shake-flask experiments, vegetative mycelium was used to inoculate 500 ml baffled Erlenmeyer flask containing 50 ml of the liquid media described above. Cultures were incubated at 28 °C on an orbital shaker at 250 rpm. *N. gerenzanensis* tended to grow as rather compact masses or pellets in a liquid media, making it impossible to measure optical density. Biomass was determined by dry weight (dw) as previously described^7^.

The antibacterial activity of *N. gerenzanensis* grown on solid media was measured by microbiological assays as previously described^42^ using *S. aureus* SA1 as a testing organism. A40926 production in liquid media was assayed by high-performance liquid chromatography (HPLC) as previously described^7^.

Quantitative assessment of phenylalanine (Phe), tyrosine (Tyr) (both from Cambridge Isotope Laboratories, Andover, MA, USA), 3,5-dihydroxyphenylglycine (Dpg) and 4-hydroxyphenylglycine (Hpg) (both from Sigma-Aldrich, St. Louis, MO, USA) was carried out by LC-MS/MS (Mallinckrodt Baker B.V., Deventer, Netherlands).

For sample preparation, 100 μL of methanol containing labelled standards ^13^Phe-C6 and ^13^Tyr-C6 (final concentration of 2.5 μmol/L) were added to 50 μl of each sample in a 1.5 mL tube. After 20 minutes at room temperature on an orbital shaking system, the samples were dried under a nitrogen flow at 65 °C. The extracted analytes were resuspended in 300 μL of acetonitrile/water (70:30, v/v) containing 0.05% of formic acid. 60 μl of the samples were injected in the flow injection analysis mode for the MS/MS experiments (3 minutes/analysis). Analyte dosage was performed in triplicate.

An API 4000 triple quadrupole mass spectrometer (Applied Biosystems-Sciex, Toronto, Canada) coupled with the Agilent high performance liquid chromatograph of the 1200 series (Agilent Technologies, Waldbronn, Germany) was for used LC-MS/MS analysis. The Turbo Spray Ion source was operated in positive ion mode with a needle potential of +5900 V. The declustering potential (DP) and collision energy (CE), Entrance Potential (EP) and Collision cell Exit Potential (CXP) were optimized for each analyte using Analyst 1.6.2 software. MS and MS/MS spectra were collected in continuous flow mode by connecting the infusion pump directly to the source. A standard solution of l μM for each analyte in methanol was infused at 10 μL/min. The multiple reaction monitoring (MRM) transitions and appropriate detection settings are presented (in the order: analyte, Q1 mass, Q3 mass, DP volts, EP volts, CE volts, CXP volts): Phenylalanine, 166.2, 120.1, 50, 10, 19, 5; ^13^Phe-C6, 172.1, 126.1, 52, 10, 18, 6; Tyrosine, 182.1, 136.1, 37, 6, 21, 7; ^13^Tyr-C6, 188.1, 142.1, 37, 6, 21, 7; 4-Hydroxy-L-phenylglycine, 168.3, 151.1, 30, 10, 12, 8; (S)-3,5-Dihydroxyphenylglycine, 184.3, 167.1, 49, 10, 19, 9.

Hpg and Dpg, for which no deuterated internal standards were available, were quantified by using the standards with the closest molecular mass (^13^Phe-C6 for 4-Hydroxy-L-phenylglycine and ^13^Tyr-C6 for (S)-3,5-Dihydroxyphenylglycine). Data were quantitatively analysed with ChemoView v1.2 software by comparing the signal intensities of the analyte and its corresponding internal standard or the standard next to the spectrum.

### Plasmids and DNA procedures

Genomic DNA was extracted from *N. gerenzanensis* and derivative strains grown in 50 ml SM medium as described previously^7^. Plasmids pTYM18, pTYM-rpoB(R) or pTYM-rpoB(R) (N426H)^11^ were introduced into *N. gerenzanensis* by intergeneric conjugation with *E. coli* GM2929/pUB307::Tn7 as described^57^. To allow plasmid selection, conjugation medium was supplemented with kanamycin (25 μg/ml). The presence of plasmids in transconjugants was confirmed by PCR and Southern blot^58^.

ΦNon1 DNA and integration of ΦNon1 DNA into the chromosomal *attB* site was analyzed by PCR using the primer pairs: 5′-TCGTCCGGGTCCATGATCGT-3′ and 5′-GTAACGCACATCACAGCTGG-3′ (*attB* empty integration site); 5′-AAGGTCACCAAGCACATGCC-3′ and 5′-GAGACGGTGGTGCTGTTCAT-3′ (ΦNon1 prophage DNA); 5′-TGACACTGGGTGACCGTTCG-3′ and 5′-GATCACGCACGTGTAACGCA-3′ (ΦNon1 circular phage DNA); 5′-ACGAGCCGACAATATTCGAC-3′ and 5′-ACGAGCCGACAATATTCGAC-3′ (ΦNon1 circular phage DNA).

### Whole genome sequencing

The *de novo* whole genome sequencing of *N. gerenzanensis* was carried out using the Genome Sequencer GS System (454 Life Sciences and Roche, Basel, Switzerland), as previously described^15, 16^. A titration run was performed to calculate the amount of library required for the emulsion PCR. Library amplification and sequencing were carried out following the manufacturer’s directions (both GS20 and GS FLX protocols). Next, 3 and 8 kb paired ends (PE) libraries were prepared and sequenced to obtain oriented contigs, according to the manufacture’s directions (454 Life Sciences and Roche, Basel, Switzerland). This procedure allows the sequence of paired reads separated by about 3 and 8 kb respectively, thus allowing contigs orientation and distance in the original genome. Each PE library was sequenced on the GS instrument (Titanium FLX Chemistry).

The resulting sequencing reads were analyzed and processed into contigs and scaffolds generating a consensus sequence by using the Roche *newbler* assembler tool (version 2.9). The *GapCloser* tool was then used (version 1.12) of the SOAPdenovo2 assembler^59^ to fill gaps of the obtained assembly. At the end of this procedure, still-present gaps in the main chromosome were filled by performing PCR amplifications followed by Sanger sequencing of each amplicon. Primer pairs were designed to test multiple contig combinations. PCR amplifications were prepared as previously described^15, 16^. Finally, in order to completely sequence and annotate the genome of *N. gerenzanensis* a specific sequencing run was designed to deeply analyze rRNA gene regions. To avoid difficulties in contig assembly due to high similarity within these genes, 3 long PCR (5,800 bp each) were carried out to amplify ribosomal genes regions not univocally assembled. Each amplicon was used to prepare a univocally tagged shotgun library sequenced in one GS Junior run following the manufacturer’s instructions (454 Life Sciences and Roche, Basel, Switzerland). The assembled pseudo-molecules were submitted to Rapid Annotation using the Subsystem Technology (RAST) annotation server for subsystem classification and functional annotation^60^. The protein coding sequences (CDSs) were assigned using BLASTp with the KEGG orthology (KO) database. tRNA was predicted with tRNAscan-SE-1.23^61^, and rRNA genes were predicted with RNAmmer 1.2^62^. Rapid identification, annotation and analysis of secondary metabolite biosynthesis gene clusters were carried out by using the antiSMASH 2.0 platform^63, 64^. The NRPSpredictor2 web server was used to predict non-ribosomal peptide synthase (NRPS) adenylation domain specificity^65^.

The genome sequence is deposited in the EMBL/GenBank under accession number: Nonomuraea_93944_v1 |PRJEB13546 |ERS1118942 |LT559118-LT559121.

### RNA extraction, sequencing and differential gene expression analysis

For each strain, RNA was extracted from mycelium pellets deriving from 1-ml culture samples using the GeneEluteTM total RNA Purification Kit (SIGMA). RNAs were quantified with a NanoDrop spectrophotometer (NanoDrop Technologies) and quality-assessed on an Agilent Bioanalyzer (Agilent Technologies). The RNA samples showing a RIN (RNA Integrity Number) higher than 7 were processed for library preparation. Totally, 6 RNA (3 for each pH value) were analyzed. RNA samples were purified to remove rRNA using the Bacteria Ribo Zero kit (Epicentre). The depleted RNAs were retrotrascribed, indexed and sequenced in one sequencing run on the MiSeq instrument (Illumina).

Sequence quality check was performed on fastq files by using FastQC version 0.10.1 (www.bioinformatics.babraham.ac.uk/projects/fastqc/). PRINSEQ version 0.20.4^66^ was used to filter out contaminants. Bowtie2 version 2.2.1^67^ was used to index genomic contigs and to align reads to these contigs. SAMtools version 0.1.19^68^ was used to convert the SAM file generated by Bowtie2 to the BAM format, which was then sorted and indexed. SAMtools was also used to perform alignment quality check. The sorted BAM file was used as input for easyRNASeq version 2.2.0^69^, along with a GTF file containing gene annotation, to obtain count tables. The count tables (File S2) were fed to DESeq2 version 1.6.2^39^ in order to perform differential gene expression analysis. Gene-set enrichment analysis was performed to investigate the differential expression of 59 gene-sets classified as clustered (C) and dispersed (D) gene-sets (File S3–S4). Clustered gene-sets are groups of genes located closely in the genome and involved in secondary metabolite production, identified by the AntiSMASH 2.0 platform^63, 64^. Dispersed gene-sets are groups of genes involved in central and intermediary metabolism, and they were manually defined by searching RAST annotation by subsystem or gene function. Gene-set enrichment analysis was performed with GSEA Pre-ranked version 2^70^. GSEA was given as input a list of log2 fold changes calculated by DESeq2, ranked by fold change value, and the gene-sets to test for differential expression, in the form of a gmt file. Gene randomization was chosen to calculate p-values, the number of random gene samplings for each gene-set was set to 1000. A PHP script was developed to produce the graphical representation of GSEA results proposed in this work.

## Electronic supplementary material

Supplementary Data

Supplementary Table S1

Supplementary Table S2

Supplementary Table S4
