# Supplementary material for: The complete 12 Mb genome and transcriptome of Nonomuraea gerenzanensis with new insights into its duplicated “magic” RNA polymerase
Source: Sci Rep. 2016 Dec 21;6:18. doi: 10.1038/s41598-016-0025-0 (PMC5431353; doi:10.1038/s41598-016-0025-0)
Supplement: Supplementary file 1 — Supplementary Data [file 41598_2016_25_MOESM1_ESM.doc]

**Supplementary Data**

**The complete 12 Mb genome and transcriptome of *Nonomuraea gerenzanensis* with new insights into its duplicated “magic” RNA polymerase**

Valeria D'Argenio^1,2,*^, Mauro Petrillo^1,3,*^, Daniela Pasanisi^4,5^, Caterina Pagliarulo^6^, Roberta Colicchio^2^, Adelfia Talà^4^, Maria Stella de Biase^2^, Mario Zanfardino^2^, Emanuela Scolamiero^1^, Chiara Pagliuca^1,2^, Antonio Gaballo^4,7^, Annunziata Gaetana Cicatiello^2^, Piergiuseppe Cantiello^1^, Irene Postiglione^1^, Barbara Naso^1^, Angelo Boccia^1^, Miriana Durante^8^, Luca Cozzuto^1^, Paola Salvatore^1,2^, Giovanni Paolella^1,2^, Francesco Salvatore^1,2, **^ and Pietro Alifano^4, **^

^1^CEINGE-Biotecnologie Avanzate, Naples, Italy; ^2^Department of Molecular Medicine and Medical Biotechnology, Federico II University Medical School, Naples, Italy; ^3^European Commission, Joint Research Centre (JRC), Ispra, Italy; ^4^Department of Biological and Environmental Sciences and Technologies (DiSTeBA), University of Salento, Lecce, Italy; ^5^Department of Biotechnology and Life Sciences, University of Insubria, Varese, Italy; ^6^Department of Sciences and Technologies, University of Sannio, Benevento, Italy; ^7^CNR NANOTEC – Institute of Nanotechnology, Center of Nanotechnology c/o Campus Ecotekne, Lecce, Italy; ^8^CNR – Institute of Sciences of Food Production (ISPA), Operative Unit of Lecce, Lecce, Italy.

^*^These authors contributed equally to this work.

^**^Corresponding authors with equal contribution and responsabilities: **F Salvatore**, CEINGE-Biotecnologie Avanzate, Via Gaetano Salvatore 486, 80145 Naples, Italy. Tel: +39 0817463133; Fax: +390817463650; E-mail: salvator@unina.it and **P Alifano**, Department of Biological and Environmental Sciences and Technologies (DiSTeBA), University of Salento, Lecce, Italy, Centro Ecotekne Pal. B - S.P. 6, Monteroni - LECCE, Italy. Tel: +39 0832298856; Fax: +390832320626; E-mail: pietro.alifano@unisalento.it.

**Figure S1:** Blast dot plots

**Figure S2:** Gene cluster 12 coding for a putative enediyne antibiotic

**Figure S3:** Gene cluster 27 coding for iterative type II polyketide synthase (PKS) and phenoxazinone synthases involved in biosynthesis of angucyclic / phenoxazinone metabolite

**Figure S4:** Gene cluster 30 involved in biosynthesis of tabtoxin-type β-lactam

**Figure S5:** Gene cluster 6 encoding coding for putative lantibiotic/bacteriocin

**Figure S6:** Gene cluster 13 encoding coding for putative lantibiotic/bacteriocin

**Figure S7:** Gene cluster 23 encoding coding for putative lantibiotic/bacteriocin

**Figure S8:** Construction of recombinant strains

**Figure S9:** Phenotype and antibiotic production

**Figure S10:** Overview of GSEA results

**Figure S11:** Overview of GSEA results

**Figure S12:** Overview of GSEA results

**Figure S13:** Overview of GSEA results

**Figure S14:** Overview of GSEA results

**Table S1:** RAST annotation (dataset)

**Table S2:** Read counts in annotated features (dataset)

**Table S3:** Gene-sets tested for differential expression

**Table S4:** Metabolic pathways (dataset)

**
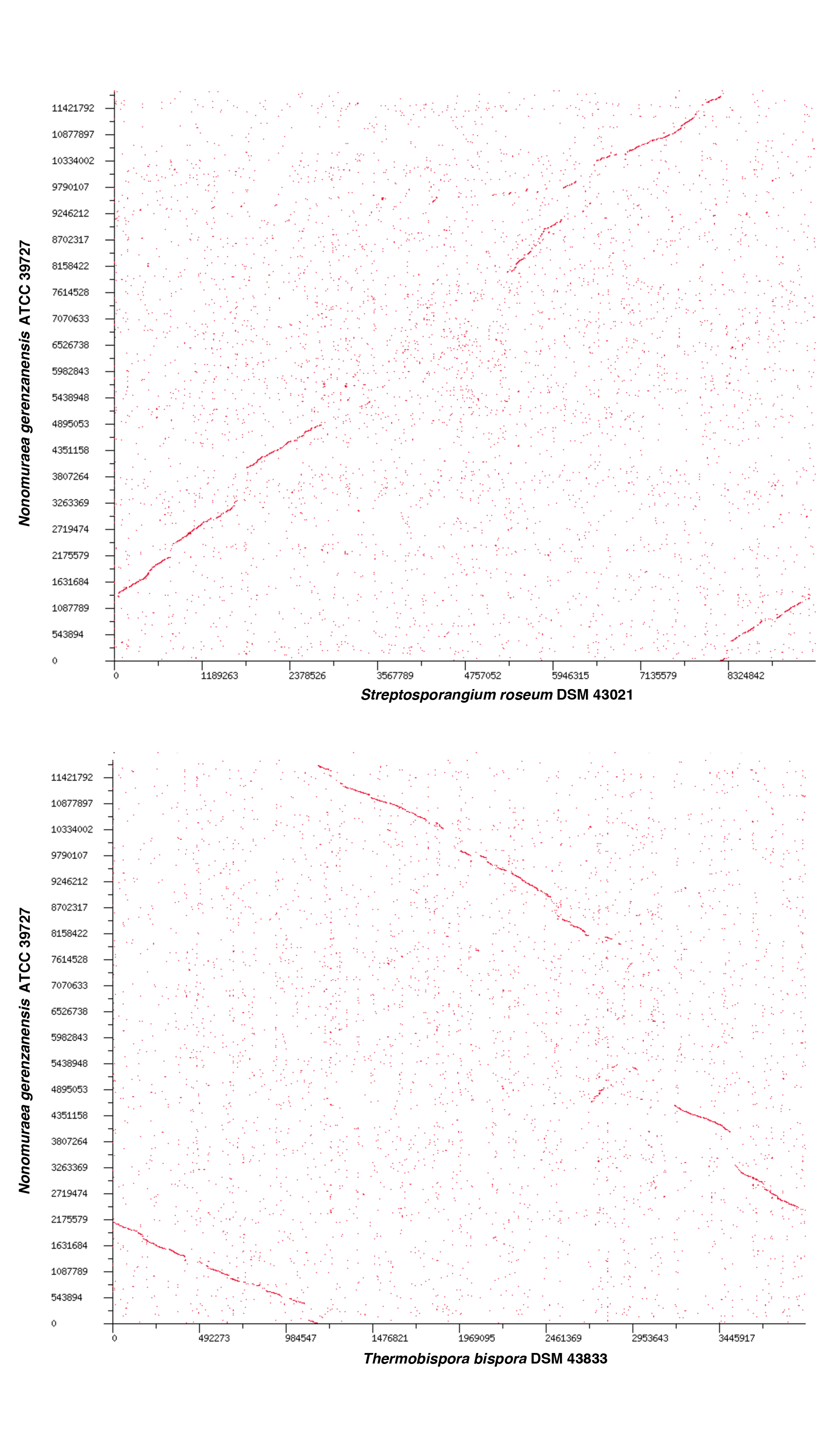
**

**Figure S1.** Blast dot plots. The high degree of conservation of gene order between *N. generzanensis* ATCC 39727, *S. roseum* DSM 43021 and *T. bispora* DSM 43833 is shown.

**
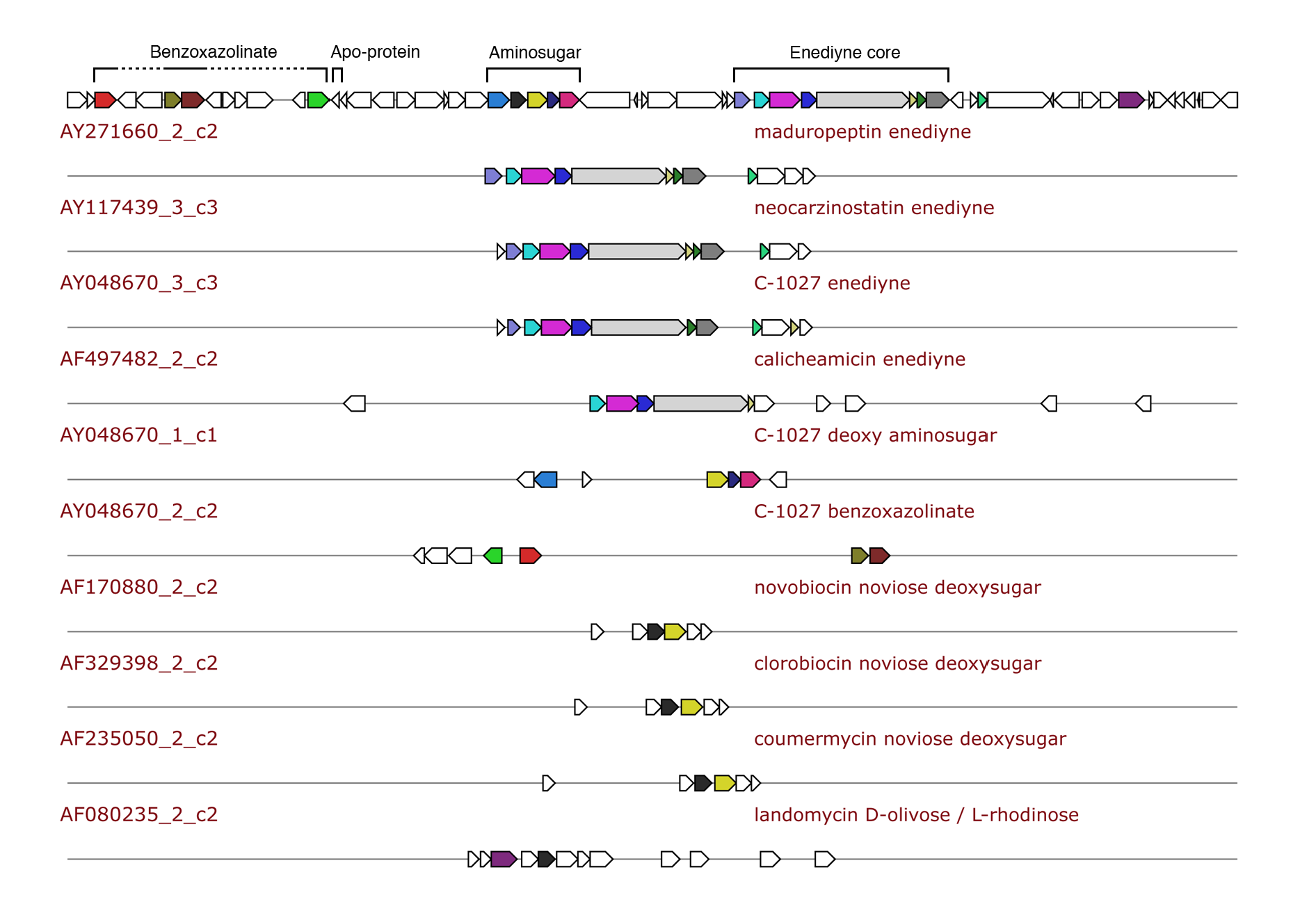
**

**Figure S2.** Gene cluster 12 coding for a putative enediyne antibiotic. The genetic map of the cluster 12 identified by antiSMASH platform is reported. Homologous clusters are shown below the map.


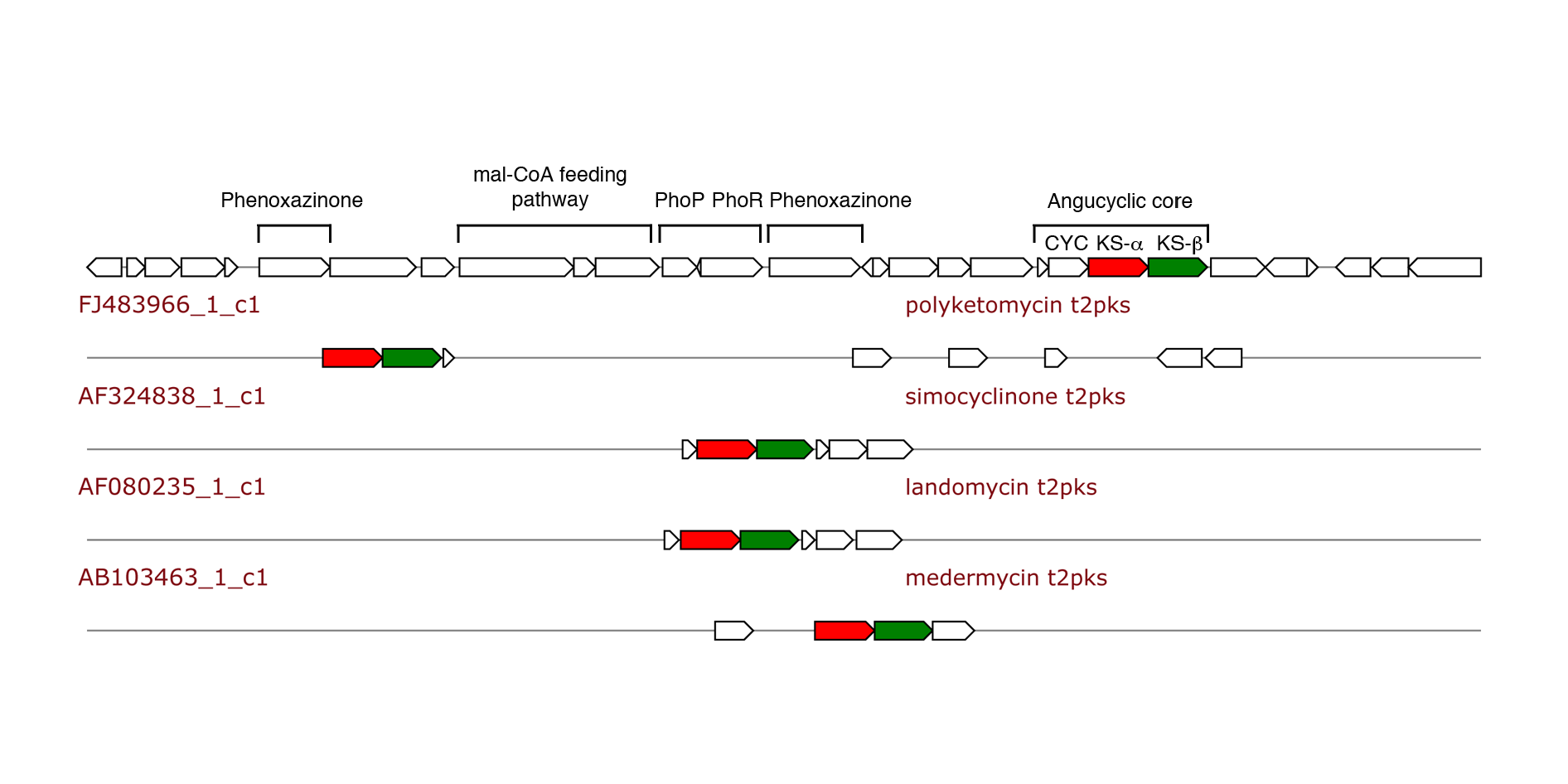


**Figure S3.** Gene cluster 27 coding for iterative type II polyketide synthase (PKS) and phenoxazinone synthases involved in biosynthesis of angucyclic / phenoxazinone metabolite. The genetic map (identified by antiSMASH) is depicted above homologous gene clusters in other microorganisms. Abbreviations indicate the following protein domains: KS-α, ketosynthase alpha subunit; KS-β, ketosynthase beta subunit; CYC, cyclase.


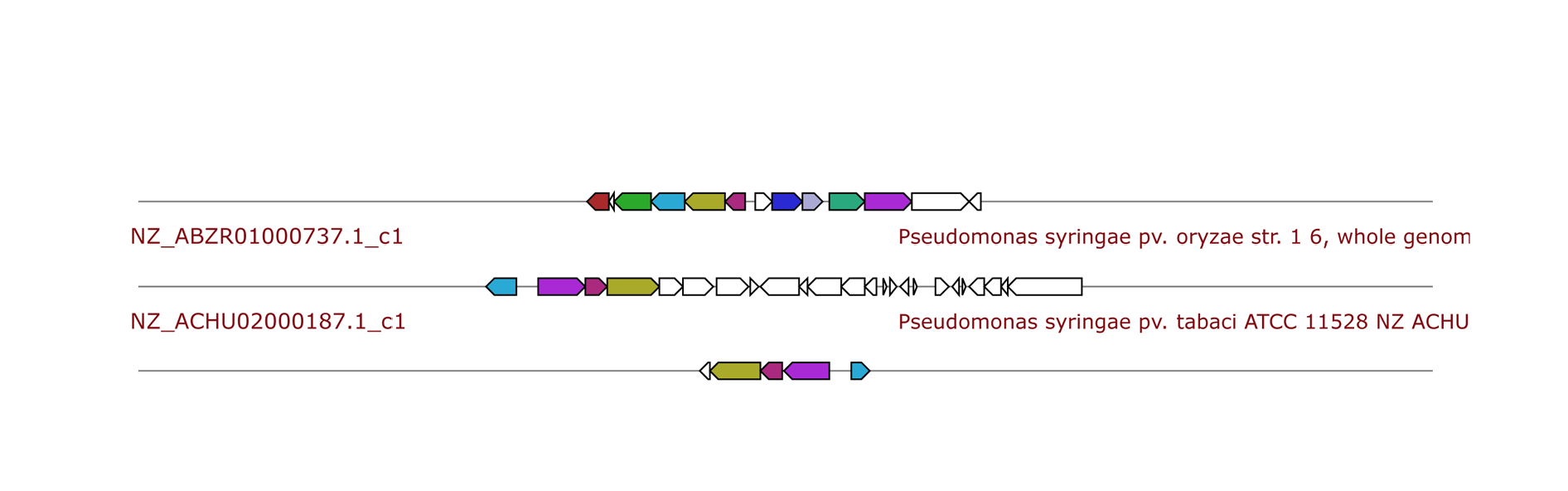


**Figure S4.** Gene cluster 30 involved in biosynthesis of tabtoxin-type β-lactam. The genetic map (identified by antiSMASH) is depicted above homologous gene clusters in phytopathogenic subspecies of *Pseudomonas syringae.*


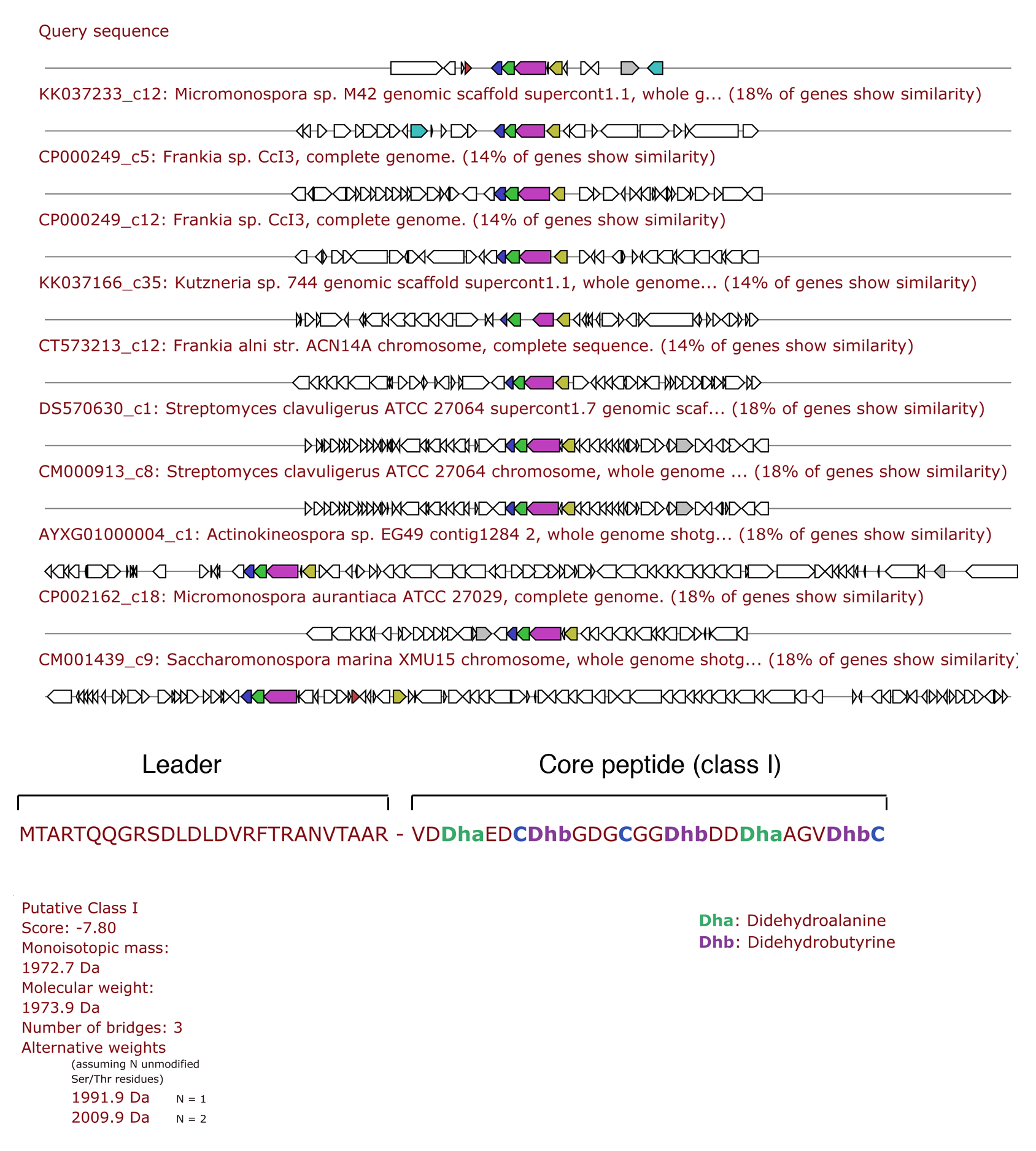


**Figure S5.** Gene cluster 6 encoding coding for putative lantibiotic/bacteriocin. The genetic map (identified by antiSMASH) is depicted above homologous gene clusters in other microorganisms (top). Predicted structures of both leader and core peptide, and molecular weight are also shown (bottom).


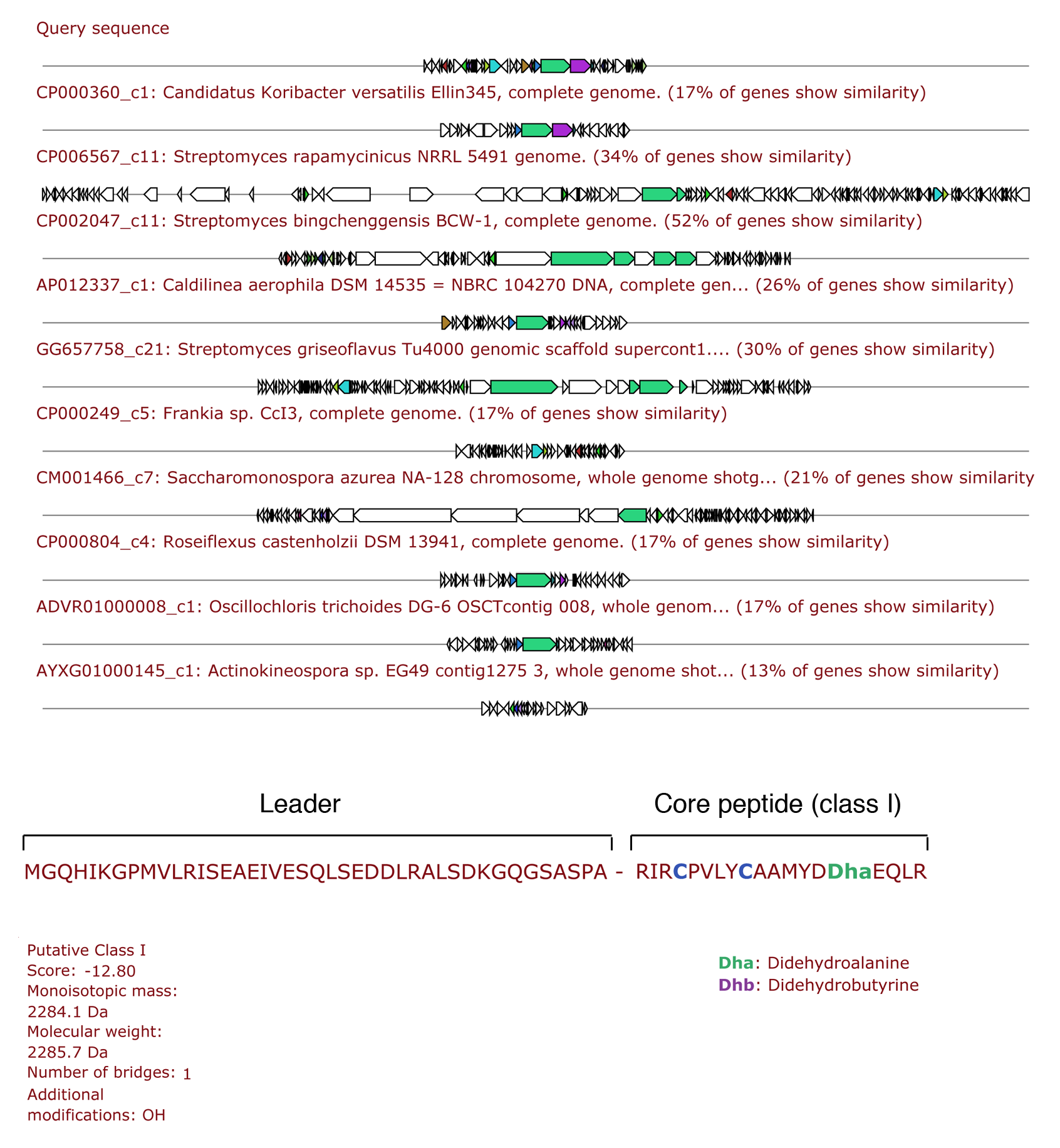


**Figure S6.** Gene cluster 13 encoding coding for putative lantibiotic/bacteriocin. The genetic map (identified by antiSMASH) is depicted above homologous gene clusters in other microorganisms (top). Predicted structures of both leader and core peptide, and molecular weight are also shown (bottom).


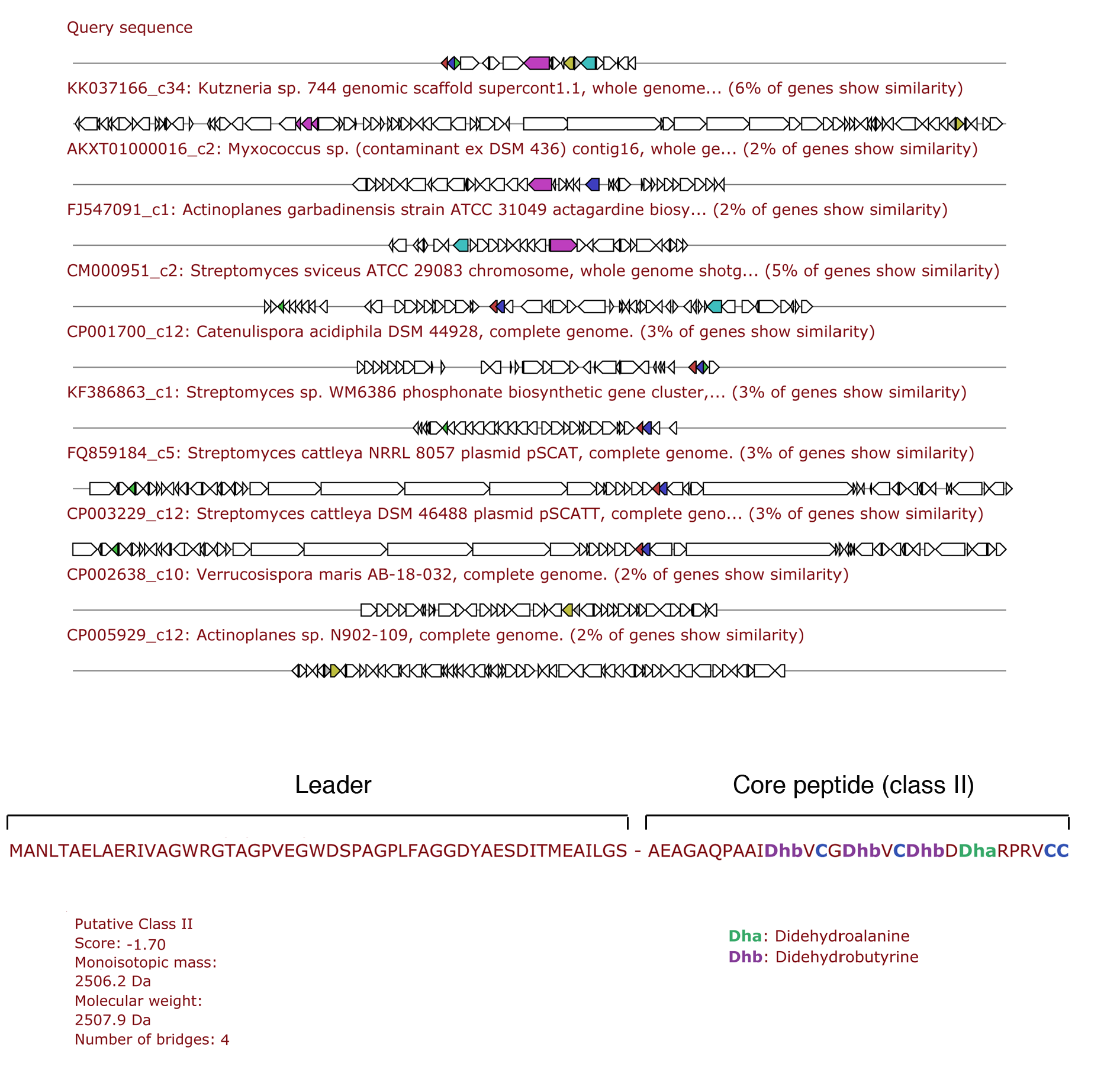


**Figure S7.** Gene cluster 23 encoding coding for putative lantibiotic/bacteriocin. The genetic map (identified by antiSMASH) is depicted above homologous gene clusters in other microorganisms (top). Predicted structures of both leader and core peptide, and molecular weight are also shown (bottom).


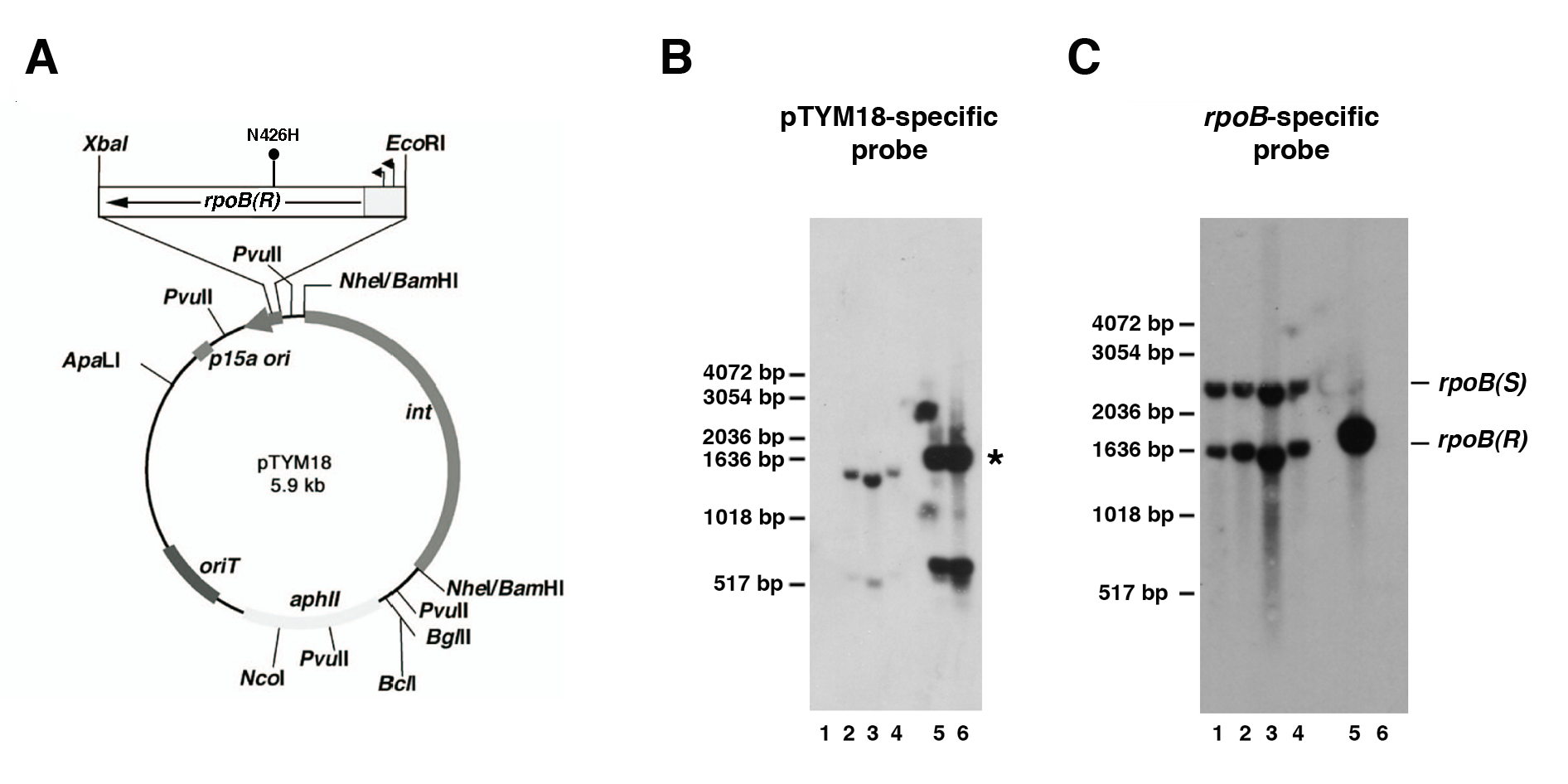


**Figure S8.** Construction of recombinant strains. *(A)* Map of pTYM18 used as a conjugative vector to transfer *rpoB(R)* or mutated *rpoB(R)^N426H^* into *N. gerenzanensis*. *p15a ori*, origin of replication in *E. coli*; *oriT*, origin of conjugative transfer; *aphII*, kanamycin resistance gene; *int*, bacteriophage ΦC31 integrase gene. *(B-C)* Southern blot analysis. Total DNA was extracted from wild strains *N.* *gerenzanensis* (lane 1) and derivative transconjugants rpoB(R) (lane 2), rpoB(R)^N426H^ (lane 4) and control (lane 3) strains, digested with *Hin*fI and analyzed by Southern blot with pTYM18-specific (panel *B*) or *rpoB*-specific probe (panel *C*). In lanes 5 and 6, *Hin*fI-digested pTYM-rpoB(R) and pTYM18 plasmid DNAs were used as control. In panel *B*, disappearance of the 1715 bp band (asterisk on the right) spanning the ΦC31 integrase gene demonstrates chromosomal integration of plasmids. In panel *C*, positions of *rpoB(S)*- and *rpoB(R)*-specific bands are indicated on the right. The positions of molecular weight ladders run in parallel are indicated on the left side of each panel.


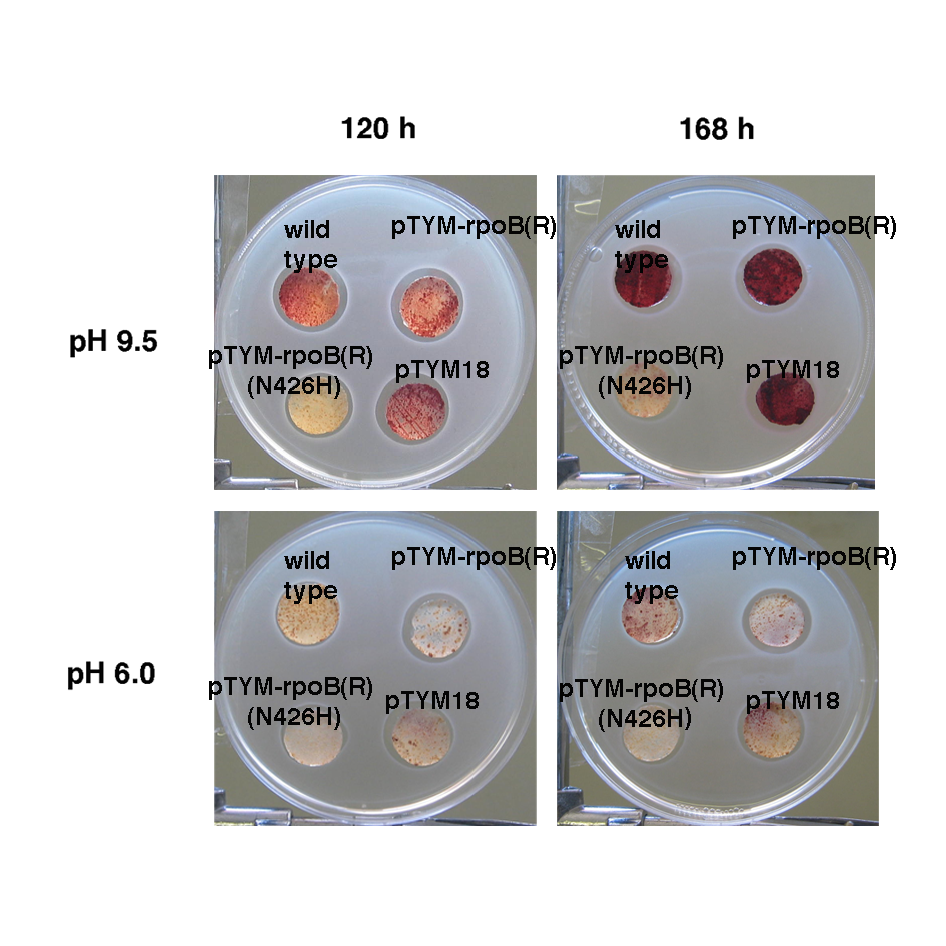


**Figure S9.** Phenotype and antibiotic production. Phenotype and antibiotic production of wild type *N.* *gerenzanensis* and derivative rpoB(R), rpoB(R)N426H and control strains (harbouring, respectively, the recombinant plasmids pTYM-rpoB(R) and pTYM-rpoB(R)(N426H), and the vector plasmid pTYM18), grown on YS agar for 120-168 h are shown. Antibiotic was assayed by microbiological assay using *S. aureus* as a tester microorganism.


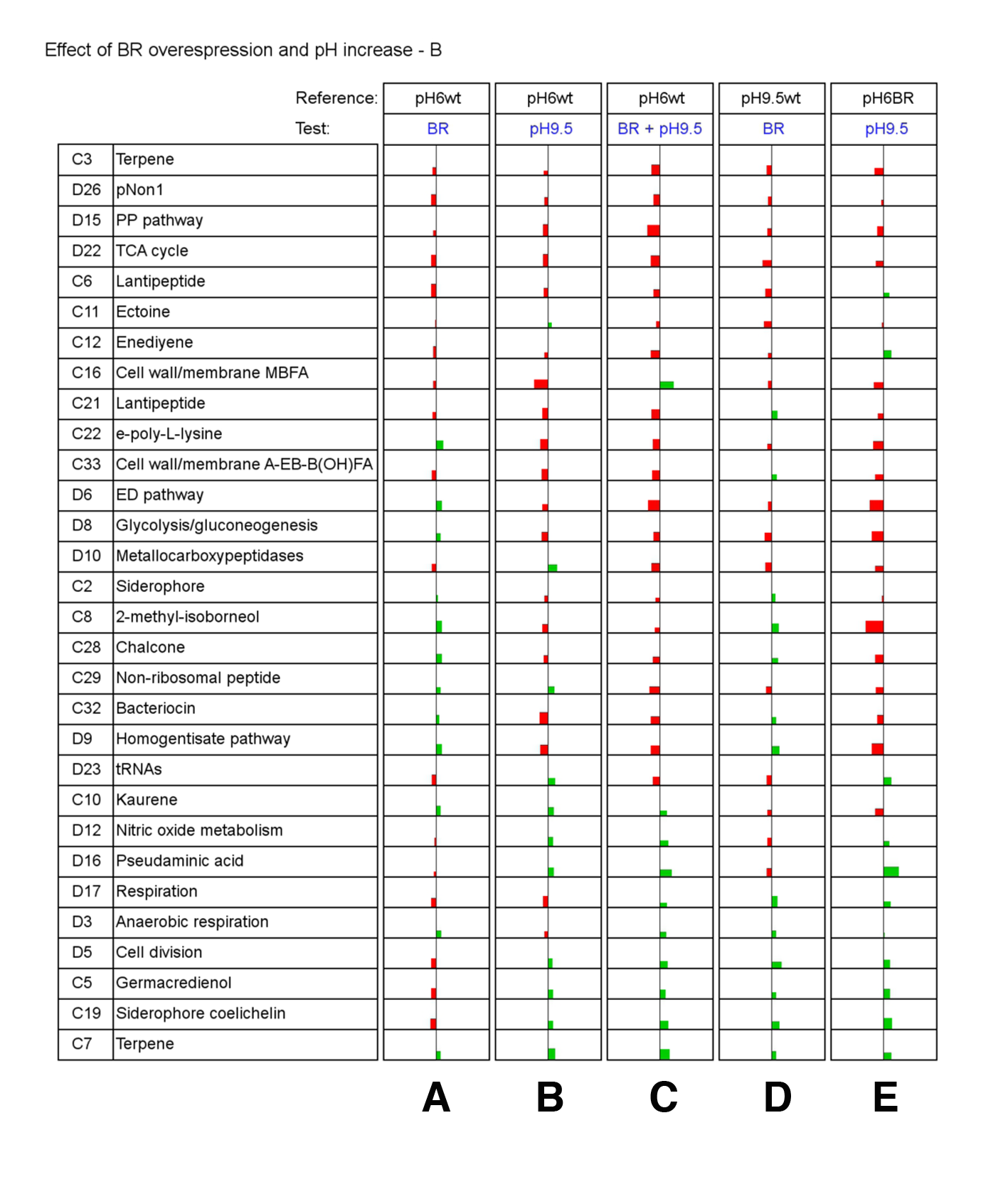


**Figure S10.** Overview of GSEA results. *(A-E)* Effects of *rpoB(R)* over-expression and pH increase are reported by contrasting rpoB(R) strain to wild type strain data. Gene-sets not passing the thresholds (NES > 1.70 and FDR < 0.1) in any of the contrasts are reported. Green and red colors indicate, respectively, up-regulation and down-regulation in test strain vs. reference strain. If a set passed these thresholds in a contrast, the background of the cell is colored in pale green. For each set in each contrast, the width of the rectangle represents the mean log_2_FC of the leading edge subset, while the height represents the NES. Gene-sets are labeled with an ID indicating whether they consist in clustered (ID number preceded by the letter C) or dispersed (ID number preceded by the letter D) genes. Abbreviations: wt, wild type strain; BR, rpoB(R) strain.


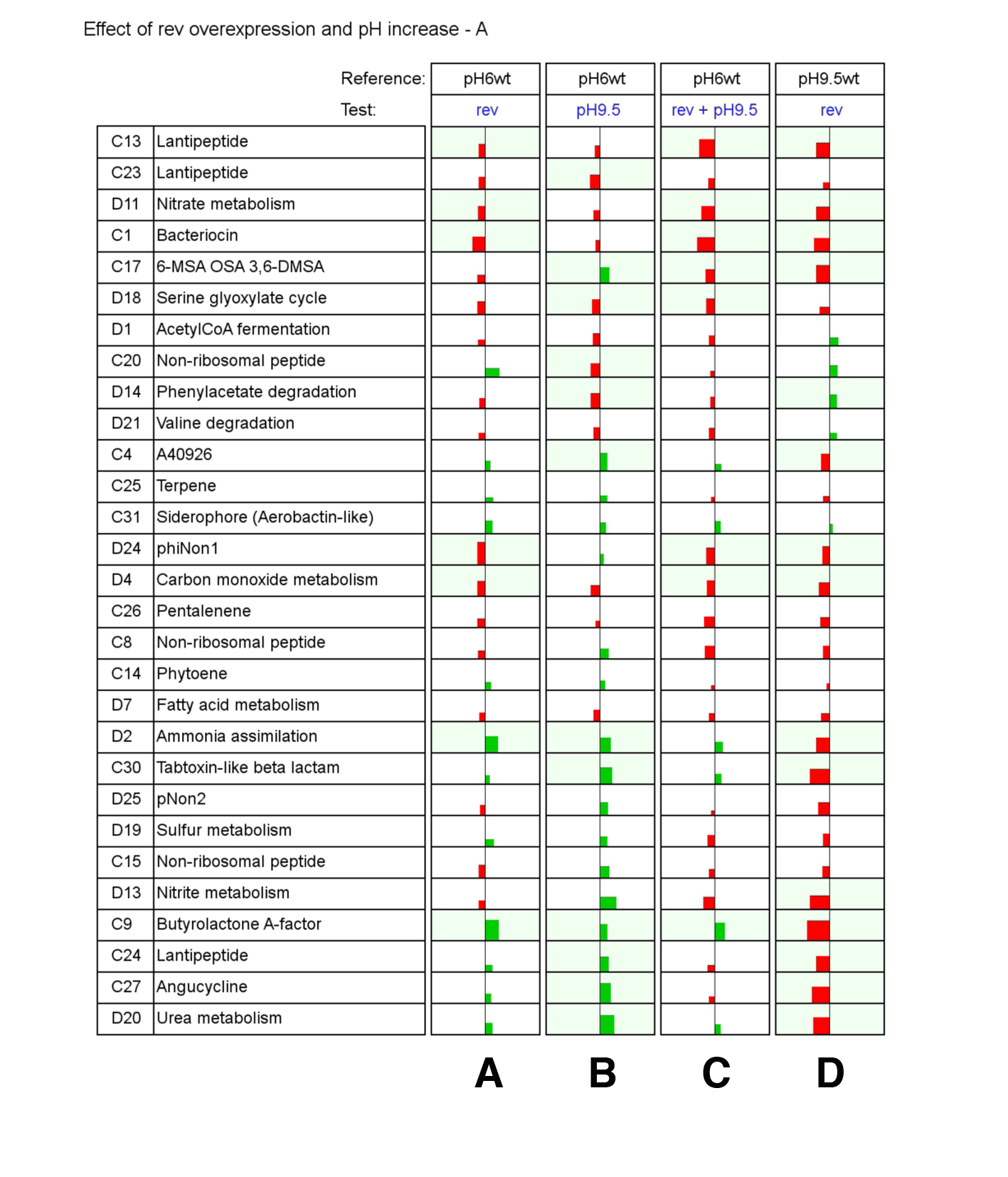


**Figure S11.** Overview of GSEA results. *(A-D)* Effects of *rpoB(R)^N426H^* expression and pH increase are reported by contrasting rpoB(R)^N426H^ strain to wild type strain data. Gene-sets with Normalized Enrichment Score (NES) > 1.70 and False Discovery Rate (FDR) < 0.1 in at least one of the contrasts are reported. Green and red colors indicate, respectively, up-regulation and down-regulation in test strain vs. reference strain. If a set passed these thresholds in a contrast, the background of the cell is colored in pale green. For each set in each contrast, the width of the rectangle represents the mean log_2_FC of the leading edge subset, while the height represents the NES. Gene-sets are labeled with an ID indicating whether they consist in clustered (ID number preceded by the letter C) or dispersed (ID number preceded by the letter D) genes. Abbreviations: wt, wild type strain; rev, rpoB(R)^N426H^ strain.


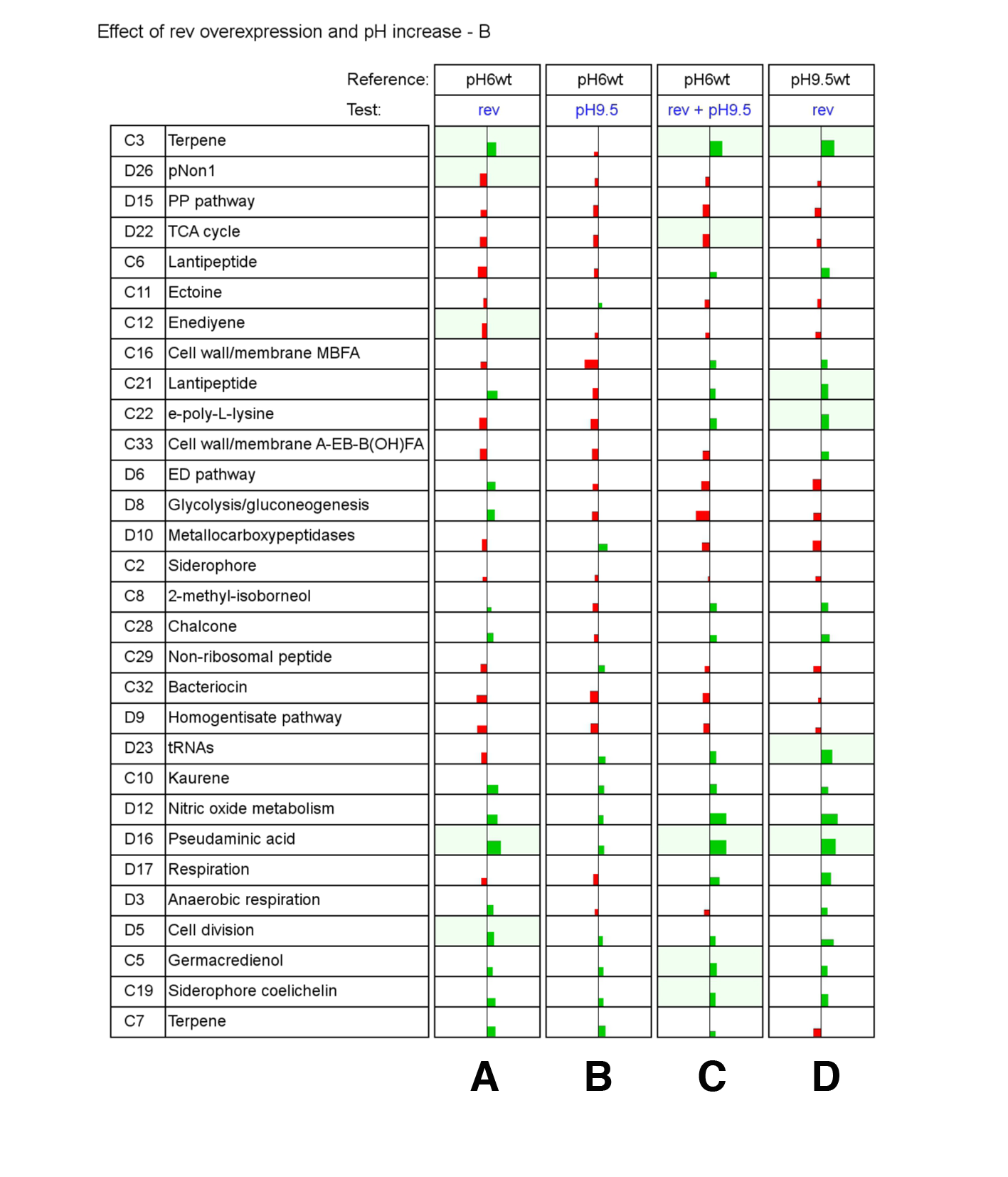


**Figure S12.** Overview of GSEA results. *(A-D)* Effects of *rpoB(R)^N426H^* expression and pH increase are reported by contrasting rpoB(R)^N426H^ strain to wild type strain data. Gene-sets not passing the thresholds (NES > 1.70 and FDR < 0.1) in any of the contrasts are reported. Green and red colors indicate, respectively, up-regulation and down-regulation in test strain vs. reference strain. If a set passed these thresholds in a contrast, the background of the cell is colored in pale green. For each set in each contrast, the width of the rectangle represents the mean log_2_FC of the leading edge subset, while the height represents the NES. Gene-sets are labeled with an ID indicating whether they consist in clustered (ID number preceded by the letter C) or dispersed (ID number preceded by the letter D) genes. Abbreviations: wt, wild type strain; rev, rpoB(R)^N426H^ strain.


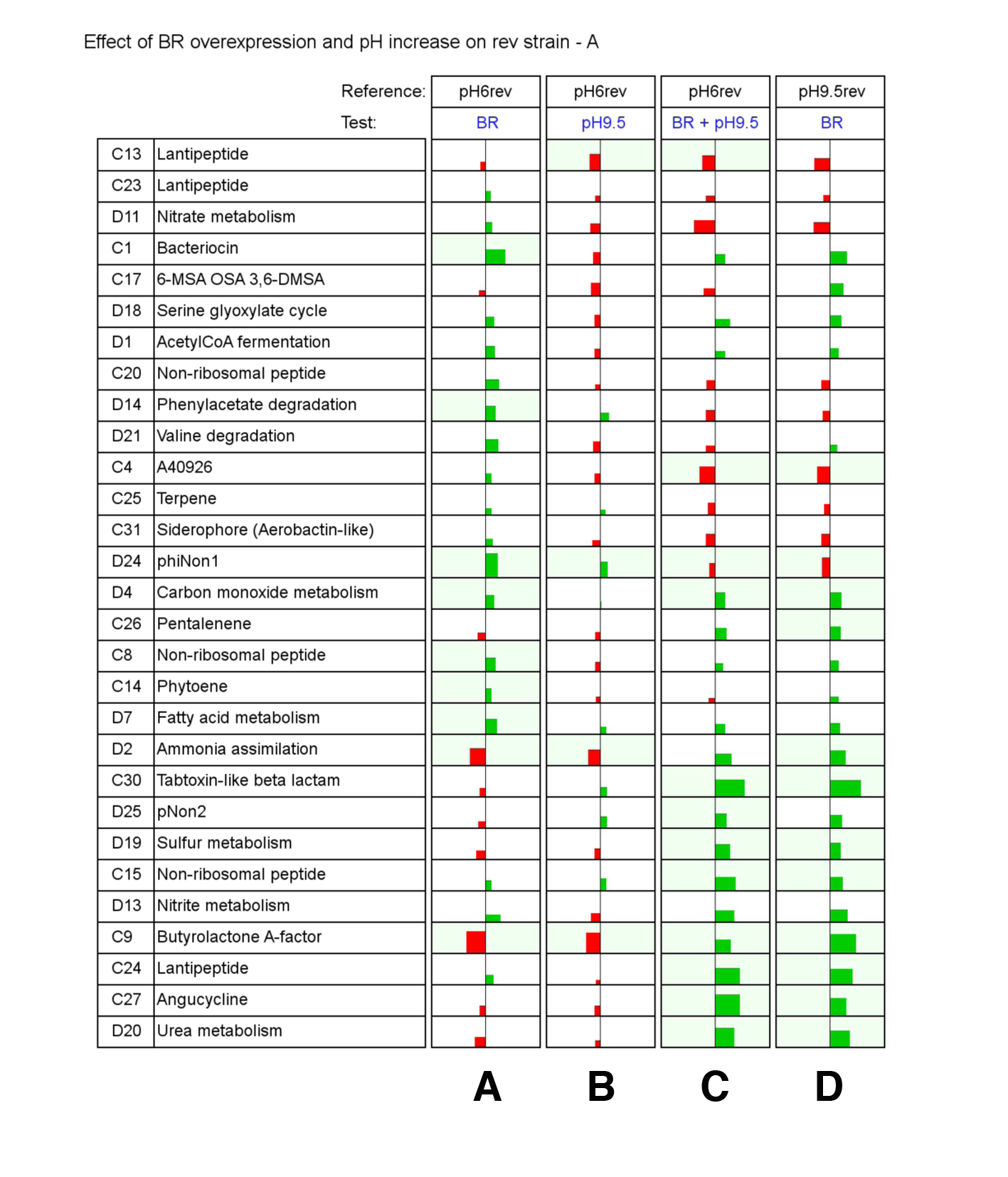


**Figure S13.** Overview of GSEA results. *(A-D)* Effects of *rpoB(R)* over-expression and pH increase are reported by contrasting rpoB(R) strain to rpoB(R)^N426H^ strain data. Gene-sets with Normalized Enrichment Score (NES) > 1.70 and False Discovery Rate (FDR) < 0.1 in at least one of the contrasts are reported. Green and red colors indicate, respectively, up-regulation and down-regulation in test strain vs. reference strain. If a set passed these thresholds in a contrast, the background of the cell is colored in pale green. For each set in each contrast, the width of the rectangle represents the mean log_2_FC of the leading edge subset, while the height represents the NES. Gene-sets are labeled with an ID indicating whether they consist in clustered (ID number preceded by the letter C) or dispersed (ID number preceded by the letter D) genes. Abbreviations: BR, rpoB(R) strain; rev, rpoB(R)^N426H^ strain.


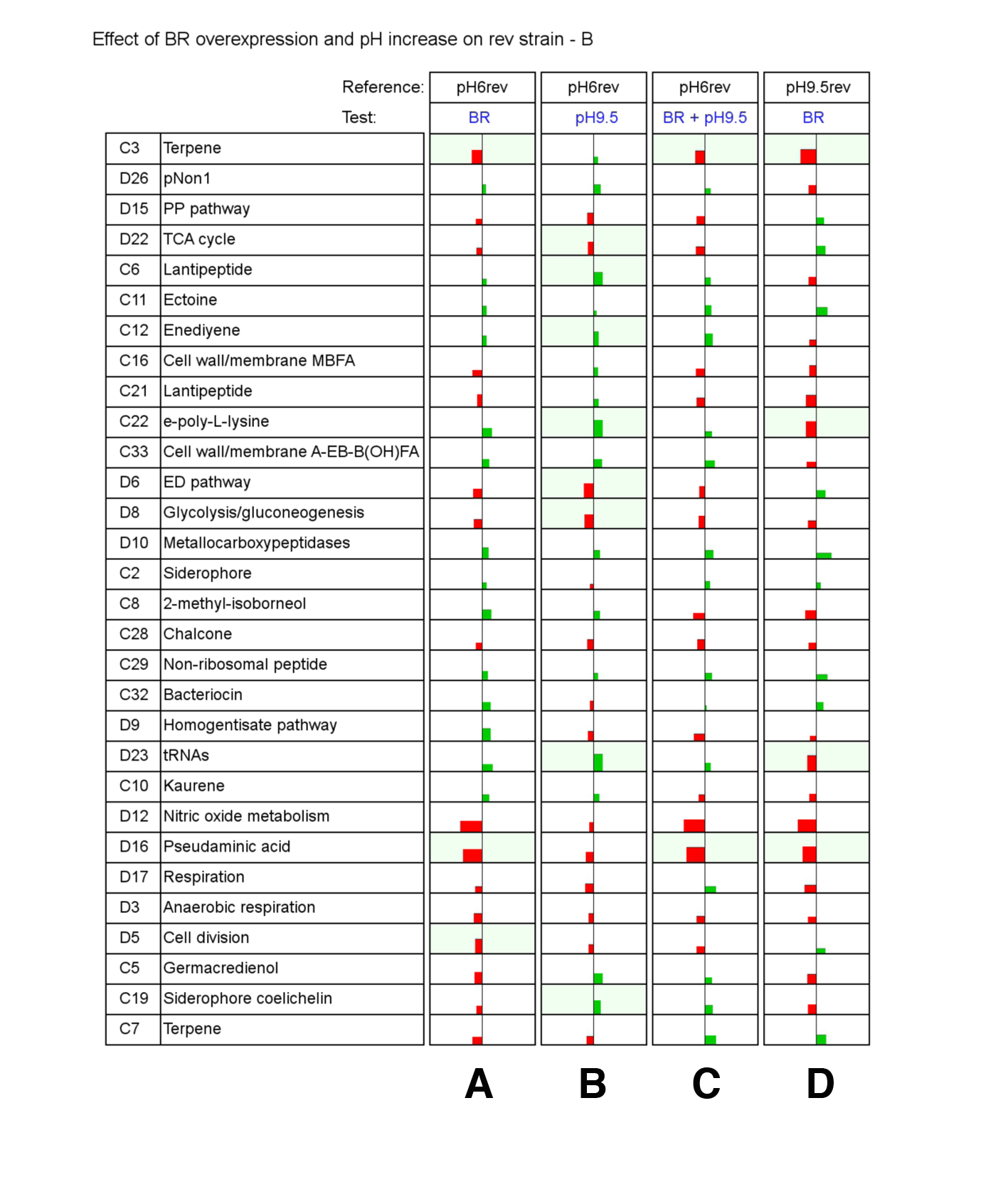


**Figure S14.** Overview of GSEA results. *(A-D)* Effects of *rpoB(R)* over-expression and pH increase are reported by contrasting rpoB(R) strain to rpoB(R)^N426H^ strain data. Gene-sets not passing the thresholds (NES > 1.70 and FDR < 0.1) in any of the contrasts are reported. Green and red colors indicate, respectively, up-regulation and down-regulation in test strain vs. reference strain. If a set passed these thresholds in a contrast, the background of the cell is colored in pale green. For each set in each contrast, the width of the rectangle represents the mean log_2_FC of the leading edge subset, while the height represents the NES. Gene-sets are labeled with an ID indicating whether they consist in clustered (ID number preceded by the letter C) or dispersed (ID number preceded by the letter D) genes. Abbreviations: BR, rpoB(R) strain; rev, rpoB(R)^N426H^ strain.

**Table S1**

**Table S2**

Please, see the xls file in which table S1 and Table S2 are more readable

| **Name** | **ID** | **N. genes** | **Source** |
| --- | --- | --- | --- |
| Bacteriocin | C1 | 6 | Antismash |
| Siderophore | C2 | 5 | Antismash |
| Terpene | C3 | 7 | Antismash |
| A40926 lypoglycopeptide | C4 | 40 | Antismash |
| Germacredienol/germacredene | C5 | 13 | Antismash |
| Lantipeptide | C6 | 8 | Antismash |
| Terpene | C7 | 4 | Antismash |
| Non-ribosomal peptide | C8 | 45 | Antismash |
| Butyrolactone A-factor | C9 | 25 | Antismash |
| Kaurene | C10 | 6 | Antismash |
| Ectoine | C11 | 10 | Antismash |
| Enediyene | C12 | 67 | Antismash |
| Lantipeptide | C13 | 28 | Antismash |
| Phytoene | C14 | 12 | Antismash |
| Non-ribosomal peptide | C15 | 40 | Antismash |
| Cell wall/membrane MBFA | C16 | 33 | Antismash |
| 6-MSA/OSA/3,6-DMSA | C17 | 41 | Antismash |
| 2-methyl-isoborbeol | C18 | 13 | Antismash |
| Siderophore coelichelin | C19 | 32 | Antismash |
| Non-ribosomal peptide | C20 | 37 | Antismash |
| Lantipeptide | C21 | 17 | Antismash |
| ε-poly-L-lysine | C22 | 20 | Antismash |
| Lantipeptide | C23 | 16 | Antismash |
| Lantipeptide | C24 | 6 | Antismash |
| Terpene | C25 | 18 | Antismash |
| Pentalenene | C26 | 18 | Antismash |
| Angucycline/Phenoxazinone | C27 | 36 | Antismash |
| Chalcone | C28 | 31 | Antismash |
| Non-ribosomal peptide | C29 | 29 | Antismash |
| Tabtoxin-like β lactam | C30 | 27 | Antismash |
| Siderophore (Aerobactin-like) | C31 | 11 | Antismash |
| Bacteriocin | C32 | 12 | Antismash |
| Cell wall/membrane MBHFA | C33 | 27 | Antismash |
| AcetylCoA fermentation | D1 | 33 | RAST |
| Ammonia assimilation | D2 | 10 | RAST |
| Anaerobic respiration | D3 | 21 | RAST |
| Carbon monoxide metabolism | D4 | 19 | RAST |
| Cell division | D5 | 63 | RAST |
| ED pathway | D6 | 32 | RAST |
| Fatty acid metabolism | D7 | 54 | RAST |
| Glycolysis/gluconeogenesis | D8 | 28 | RAST |
| Homogentisate pathway | D9 | 32 | RAST |
| Metallocarboxypeptidases | D10 | 31 | RAST |
| Nitrate metabolism | D11 | 20 | RAST |
| Nitric oxide metabolism | D12 | 4 | RAST |
| Nitrite metabolism | D13 | 8 | RAST |
| phiNon1 | D24 | 85 | RAST |
| pNon1 | D26 | 9 | RAST |
| pNon2 | D25 | 57 | RAST |
| Phenylacetate degradation | D14 | 8 | RAST |
| PP pathway | D15 | 17 | RAST |
| Pseudaminic acid | D16 | 8 | RAST |
| Respiration | D17 | 68 | RAST |
| Serine glyoxylate cycle | D18 | 61 | RAST |
| Sulfur metabolism | D19 | 108 | RAST |
| TCA cycle | D22 | 31 | RAST |
| Urea metabolism | D20 | 23 | RAST |
| Valine degradation | D21 | 41 | RAST |
| tRNA | D23 | 70 | RAST |

**Table S3.** Gene-sets tested for differential expression.

Each gene-set has an ID indicating whether it is a clustered (C) or dispersed (D) gene-set. Clustered gene-sets were defined by Antismash, a platform for identification of secondary metabolite clusters; dispersed gene-sets were defined by searching RAST results by subsystem or gene function. Colours indicate the type of gene-set: secondary metabolism (green), central/intermediary metabolism (yellow), genes located on an extrachromosomal element (purple) and tRNA genes (orange).

**Table S4**

Please, see the xls file in which table S1 and Table S2 are more readable
